# Bringing It All Together: Multi-species Integrated Population Modelling of a Breeding Community

**DOI:** 10.1007/s13253-017-0279-4

**Published:** 2017-04-18

**Authors:** José J. Lahoz-Monfort, Michael P. Harris, Sarah Wanless, Stephen N. Freeman, Byron J. T. Morgan

**Affiliations:** 1 School of BioSciences, The University of Melbourne, Parkville, VIC 3010 Australia; 2 Centre for Ecology and Hydrology, Bush Estate, Penicuik, Midlothian EH26 0QB UK; 3 Centre for Ecology and Hydrology, Maclean Building, Crowmarsh Gifford, Wallingford, Oxfordshire OX10 8BB UK; 4 National Centre for Statistical Ecology, School of Mathematics, Statistics and Actuarial Science, University of Kent, Canterbury, Kent CT2 7FS UK

**Keywords:** Bayesian inference, Long-term monitoring, Mark-resight-recovery, State-space model, Survival, Synchrony

## Abstract

Integrated population models (IPMs) combine data on different aspects of demography with time-series of population abundance. IPMs are becoming increasingly popular in the study of wildlife populations, but their application has largely been restricted to the analysis of single species. However, species exist within communities: sympatric species are exposed to the same abiotic environment, which may generate synchrony in the fluctuations of their demographic parameters over time. Given that in many environments conditions are changing rapidly, assessing whether species show similar demographic and population responses is fundamental to quantifying interspecific differences in environmental sensitivity and highlighting ecological interactions at risk of disruption. In this paper, we combine statistical approaches to study populations, integrating data along two different dimensions: across species (using a recently proposed framework to quantify multi-species synchrony in demography) and within each species (using IPMs with demographic and abundance data). We analyse data from three seabird species breeding at a nationally important long-term monitoring site. We combine demographic datasets with island-wide population counts to construct the first multi-species Integrated Population Model to consider synchrony. Our extension of the IPM concept allows the simultaneous estimation of demographic parameters, adult abundance and multi-species synchrony in survival and productivity, within a robust statistical framework. The approach is readily applicable to other taxa and habitats.

Supplementary materials accompanying this paper appear on-line.

**Electronic Supplementary Material:**

Supplementary materials for this article are available at 10.1007/s13253-017-0279-4.

## Introduction

Understanding population dynamics and trends is critical for the management of species, be they threatened, invasive or harvested. A long tradition exists of monitoring wildlife population abundance and the demographic rates that drive its fluctuation, with statistical approaches developed to help scientists and managers understand the environmental drivers of change in these parameters (Williams et al. 2002). Of special relevance for our study are long-term monitoring programs, which can act as essential ecosystem sentinels for environmental change and provide the time-scale required for an improved understanding of the relationships between demography, population and environment at a multi-decadal scale, particularly for long-lived species (Wooller et al. 1992).

Long-term wildlife studies are often intensive and generate a wealth of data. When population counts and demography-related data are collected, these data can be analysed together in a single Integrated Population Model (IPM; Besbeas et al. 2002). By constructing a single likelihood function from all the data on abundance and demography, the IPM estimates are a compromise across the various sources of information, often achieving improved parameter estimation or allowing estimation of parameters that could not be obtained from the datasets in isolation (e.g. productivity: Besbeas et al. 2002, immigration: Abadi et al. 2010). Various types of IPMs have been proposed to date, corresponding to a variety of sources of data, but they have traditionally dealt with a single species.

Species do not occur in isolation but exist within communities and ecosystems. Expanding the IPM framework by incorporating multiple species allows new questions of ecological interest to be addressed, by modelling the association or direct interaction between species. The only example of multi-species IPM to date (Péron and Koons 2012) models competition between two species; predator–prey interactions could be studied with a similar structure. A related ecological question is synchrony. Sympatric species are exposed to the same abiotic environment, and this common exposure may generate synchrony in some aspect of these species’ response to their common environment, including population trends and the temporal variation of demographic parameters. The study of synchrony (and asynchrony) is relevant to understanding community structure and its response to environmental changes, and can provide clues to guide further research (McCarthy 2011). Traditional approaches to modelling multi-species synchrony usually involve pairwise species comparisons. An alternative approach, based on a truly multi-species view, was recently proposed to partition the between-year variance in a demographic parameter into a ‘synchronous’ component (common to all species within a set), and species-specific ‘asynchronous’ components, as well as to estimate the proportion of each component accounted for by environmental covariates (Lahoz-Monfort et al. 2013, 2014; Swallow et al. 2016). In this paper, we combine data integration in two different conceptual dimensions: across demography for each species (IPM) and across species to estimate multi-species synchrony, thus extending the traditional concept of single-species IPM (ssIPM) in the first multi-species IPM (msIPM) defined to estimate synchrony.

Our data come from the Isle of May, Scotland ($56^{\circ }11^{\prime }$N, $2^{\circ }34^{\prime }$W), one of the four ‘Key Site’ seabird colonies in UK’s Seabird Monitoring Programme, where detailed monitoring of abundance, breeding success and adult survival of various seabird species is carried out. We construct a multi-species IPM for three alcid species: the Atlantic puffin *Fratercula arctica*, the common murre (or guillemot) *Uria aalge* and the razorbill *Alca torda*; hereafter puffin, murre and razorbill, respectively. We start by describing the structure of independent ssIPMs for each species. These are then modelled jointly, estimating population abundance, demographic parameters including survival and productivity, and multi-species synchrony in these parameters, in a robust way. The datasets used in this study can be obtained from the first author by request.

## Single-Species Integrated Population Models (ssIPM)

Methodologies used to collect field data are described elsewhere (Harris and Wanless 1988, 1989, 2011; Harris et al. 2015): mark-resight data of individuals marked as breeding adults of unknown age, total counts of chicks leaving the colony (referred to as fledged even though murre and razorbill chicks are flightless when they leave) from a number of monitored nests, and colony-wide counts of breeding pairs, conducted annually for murres and razorbills and less frequently for puffins. For murres, datasets are also available on the proportions of breeding pairs that skipped breeding in different years and mark-resight-recovery data from individuals banded as chicks, which contributes valuable information regarding immature survival and pre-recruitment emigration. We use data from 1984 to 2009 and denote the $T=26$ years of data by $t=1,{\ldots },T$. We use parameter subscripts (or superscripts in likelihood functions) to identify species (razorbill: *R*; puffin: *P*; murre: *M*) and *S* when describing general model structures to refer to any species within a set. The following sections describe the specific datasets and single-species IPMs (ssIPMs) for the three species, with a detailed account of parameters involved and their relationship through the IPM.

### Breeding Success Data

Breeding success data (Reed et al. 2015) consist of a series of yearly counts of chicks $C_S \left( t\right)$ that fledge from a number of monitored marked adult pairs $E_S \left( t\right)$ that make a breeding attempt. As all three species lay a single egg, data can be modelled as a binomial variable $C_S \left( t\right) \sim \hbox {bin}\left( {E_S \left( t\right) ,\rho _S \left( t \right) }\right)$, where $\rho _S \left( t\right)$ is the productivity of species S, in year *t*. We represent the data using vectors ${\varvec{C_S}} =\left\{ {{{C_S}} \left( t\right) :t=1,\ldots , T} \right\}$ and ${\varvec{E_S}} =\left\{ {{{E_S}} \left( t \right) :t=1,\ldots , T} \right\}$, and the full dataset as ${\varvec{P_S}} =\left\{ {{\varvec{C_S, E_S}}} \right\}$. Letting ${\varvec{\rho _S}} =\left\{ {\rho _S \left( t \right) :t=1,\ldots , T} \right\}$ be the set of year-specific productivity parameters, the likelihood corresponding to the binomial model for a breeding success (‘BS’) dataset is

$$\begin{aligned} L_{\mathrm{BS}}^S \left( {{\varvec{P_S}} |{\varvec{\rho _S}}}\right) =\mathop {\prod }\limits _{t=1}^T \left( {{ \begin{array}{l} {E_S \left( t\right) } \\ {C_S \left( t\right) } \\ \end{array}}}\right) \rho _S \left( t\right) ^{C_S \left( t\right) }\left\{ {1-\rho _S \left( t \right) } \right\} ^{E_S \left( t\right) -C_S \left( t\right) }. \end{aligned}$$

The annual number of monitored pairs ranged from 73 to 194 pairs (mean $=$ 135) for razorbills, 32 to 196 pairs (mean $=$ 159; only 1984 and 1985 had fewer than 100 burrows monitored) for puffins, and 656 to 1014 (mean $=$ 828) for murres.

### Non-breeding Data (Murres)

Every year, a small proportion of murre pairs (typically below 10%) do not lay an egg. Non-breeding has been monitored at the Isle of May by counting the number of murres $\xi _{bM} \left( t\right)$ that do not skip breeding at any particular year *t*, out of a number of monitored individuals $\xi _{mM} \left( t\right)$ which ranged between 155 and 389 (mean = 310 murres/year). Given this dataset ${\varvec{\xi _M}} =\left\{ {\xi _{bM} \left( t\right) , \xi _{mM} \left( t \right) :t=1,\ldots , T} \right\}$, the non-breeding process can be modelled with a binomial distribution, $\xi _{bM} \left( t\right) \sim \hbox {bin}\left( {\xi _{mM} \left( t\right) ,B\left( t \right) }\right)$, where $B\left( t\right)$ is the probability of a pair breeding in year *t*. Letting ${\varvec{B}}=\left\{ {B\left( t\right) : t=1,\ldots , T} \right\}$, the likelihood for this ‘non-breeding’ model is

$$\begin{aligned} L_{\mathrm{NB}}^M \left( {{\varvec{\xi _M}} |{\varvec{B}}}\right) =\mathop {\prod }\limits _{t=1}^T \left( {{ \begin{array}{l} {\xi _{mM} \left( t\right) } \\ {\xi _{bM} \left( t\right) } \\ \end{array}}}\right) B\left( t\right) ^{\xi _{bM} \left( t\right) }\left\{ {1-B\left( t \right) } \right\} ^{\xi _{mM} \left( t\right) -\xi _{bM} \left( t \right) }. \end{aligned}$$

A small number of razorbill and puffin pairs also do not breed in a given season, but data are not available to model these processes.

### Mark-Resight Data: Adult Survival

Between 1984 and 2009, 163 breeding razorbills, 578 breeding puffins and 837 breeding murres were individually colour-banded and remained individually identifiable throughout their lives. Searches were made for these birds in subsequent years. The resulting adult Mark-Resight dataset MR(A), ${\varvec{m_S}}$ for each species *S*, is modelled using the open-population Cormack–Jolly–Seber (CJS) model (reviewed e.g. in McCrea and Morgan 2015), which estimates year-dependent survival and resight probabilities. We assumed no adult emigration (estimated parameters are thus true survival) and fully year-dependent survival probabilities, ${\varvec{s_{aS}}} =\left\{ {s_{aS} \left( t\right) :t=1,\ldots , T-1} \right\}$. Based on a previous analysis (Lahoz-Monfort et al. 2011), we use year-specific resight probability ${\varvec{p}_{\varvec{S}}^*} =\left\{ {p_S^*\left( t\right) :t=1,\ldots ,T-1} \right\}$ and account for whether an individual was resighted the season before (1-year ‘trap dependence’, with constant $a_S$). Full details of the MR model and its multinomial likelihood $L_{\mathrm{MR}\left( \mathrm{A}\right) }^S \left( {{\varvec{m_S}} |{\varvec{s_{aS}}}, {\varvec{p}_{\varvec{S}}^*}, a_S}\right)$ are given in McCrea and Morgan (2015).

### Mark-Resight-Recovery Data: Juvenile Survival (Murres)

A total of 6569 murre chicks were banded between 1984 and 2009 (annual totals: 113–325; mean: 253). Large-scale banding and resighting of puffin and razorbill chicks were not possible due to logistical constraints. Each murre chick was given a unique colour-band on one leg (with an individual code) and a numbered hard metal band on the other. Two areas were used: a 400-m length of cliff (‘area A’) and a nearby skerry (‘area B’) of lesser visibility (where banding of 1356 chicks occurred only until 1997). Full details about field methods are given in Harris et al. (2007). From 1985 to 2010, regular searches were made during the breeding season for banded murres that had returned to the Isle of May. This resulted in 11,388 individual resightings (excluding initial capture but otherwise including birds seen more than once in a breeding season) which translated into 4738 detections in the mark-resight history (raw resightings include birds seen more than once in a season). In addition, 248 banded murres were reported dead elsewhere which allowed us to estimate true survival and fidelity separately, as opposed to apparent survival (their combined effect) in MR studies (Burnham 1993).

We construct the likelihood corresponding to the chick mark-resight-recovery data ‘MRR(C)’ with a generic age- and year-dependence structure, based on a computationally efficient multi-state approach with sufficient statistic matrices (McCrea 2012). We define two mutually exclusive states: State $r = 1$ (‘Isle of May’; recruited to this population); State $r = 0$ (‘Emigrated’; recruited into another breeding colony). Birds in State 0 (unobserved) do not contribute to population abundance at the Isle of May, although bands can be recovered from dead birds in this state. For resightings of murres aged $a=1,\ldots ,A$ during years $t=1,\ldots ,T$ (no recoveries after $t=T$), we can define the model parameters (dropping species subscript M for brevity):

(i) $\phi _{a,t} \left( r\right) :$ probability that a bird in state $r=\left\{ {0,1} \right\}$ aged *a* at year *t* survives until age $a+1$. We assume same survival for any state: $\phi _{a,t} \left( 1\right) =\phi _{a,t} \left( 0 \right) =\phi _{a,t}$;
(ii) $\psi _{a,t} \left( {r,s}\right) :$ probability that a bird in state $r=\left\{ {0,1} \right\}$ aged *a* in year *t*, moves to state $s=\left\{ {0,1} \right\}$ by age $a+1$, given that it is alive at this age. Fidelity is $\psi _{a,t} \left( {1,1}\right) =F_{a,t}$ and permanent emigration is $\psi _{a,t} \left( {1,0}\right) =1-F_{a,t}$. Also, $\psi _{a,t} \left( {0,1}\right) =0, \psi _{a,t} \left( {0,0}\right) =1$;
(iii) $p_{a,t} \left( r\right) :$ probability that a bird alive in state $r=\left\{ {0,1} \right\}$ aged *a* at year *t* is resighted at this age. As birds that emigrate permanently cannot be resighted, $p_{a,t} \left( 0\right) =0$. We denote resightings at the Isle of May as $p_{a,t} \left( 1\right) =p_{a,t}$;
(iv) $$\lambda _{a,t} \left( r\right) :$$ ‘reporting’ probability, i.e. probability that a bird in state $$r=\left\{ {0,1} \right\} $$ aged *a* at year *t* that dies before age $$a+1$$ is recovered dead and its numbered metal band reported (before age $$a+1)$$. We assume $$\lambda _{a,t} \left( 1\right) =\lambda _{a,t} \left( 0\right) =\lambda _{a,t}$$.

Based on McCrea (2012), we define the following probabilities, for our particular case:

(i) $Q_{a,b,t} \left( {r,s}\right) :$ probability that a bird migrates from state $r=\left\{ {0,1} \right\}$ aged *a* at year *t*, to state $s=\left\{ {0,1} \right\}$ at age $b+1$ and is unobserved between these ages:

$$\begin{aligned} Q_{a,b,t} \left( {1,0}\right)= & {} \left\{ {{ \begin{array}{ll} {\phi _{a,t} \left( {1-F_{a,t}}\right) ,} &{}\quad {a=b} \\ {\phi _{a,t} \left\{ {\left( {1-F_{a,t}}\right) Q_{a+1,b,t+1} \left( {0,0}\right) +F_{a,t} \left( {1-p_{a+1,t+1}}\right) Q_{a+1,b,t+1} \left( {1,0}\right) } \right\} ,} &{} \quad {a<b} \\ \end{array}}} \right. \\ Q_{a,b,t} \left( {1,1}\right)= & {} \left\{ {{ \begin{array}{ll} {\phi _{a,t} F_{a,t},} &{} \quad {a=b} \\ {\phi _{a,t} F_{a,t} \left( {1-p_{a+1,t+1}}\right) Q_{a+1,b,t+1} \left( {1,1}\right) ,} &{} \quad {a<b} \\ \end{array}}} \right. \\ Q_{a,b,t} \left( {0,0}\right)= & {} \left\{ {{ \begin{array}{ll} {\phi _{a,t},} &{}\quad {a=b} \\ {\phi _{a,t} Q_{a+1,b,t+1} \left( {0,0}\right) ,} &{}\quad {a<b} \\ \end{array}}} \right. \\ Q_{a,b,t} \left( {0,1}\right)= & {} 0. \end{aligned}$$

(ii) $O_{a,b,t} \left( {r,s}\right) :$ probability that a bird in state $r=\left\{ {0,1} \right\}$ aged *a* at year *t*, remains unobserved until it is resighted at age $b+1$ in state $s=\left\{ {0,1} \right\}$:

$$\begin{aligned} O_{a,b,t} \left( {1,1}\right)= & {} Q_{a,b,t} \left( {1,1}\right) p_{b+1,t+b-a+1}\\ O_{a,b,t} \left( {1,0}\right)= & {} O_{a,b,t} \left( {0,1}\right) =O_{a,b,t} \left( {0,0}\right) =0. \end{aligned}$$

(iii) $D_{a,b,t} \left( r\right) :$ probability that a bird is recovered dead between ages *b* and $b+1$, given that it was last observed alive in state $r=\left\{ {0,1} \right\}$ aged *a* at time *t*:

$$\begin{aligned} D_{a,b,t} \left( 1\right)= & {} \left\{ {{ \begin{array}{ll} {\left( {1-\phi _{a,t}}\right) \lambda _{a,t},} &{}\quad {a=b} \\ {\left( {1-\phi _{b,t+b-a}}\right) \lambda _{b,t+b-a} \left\{ {Q_{a,b-1,t} \left( {1,0}\right) +\left( {1-p_{b,t+b-a}}\right) Q_{a,b-1,t} \left( {1,1}\right) } \right\} ,} &{} \quad {a<b} \\ \end{array}}} \right. \\ D_{a,b,t} \left( 0\right)= & {} 0. \end{aligned}$$

(iv) $\chi _{a,t} \left( r\right) :$ probability that a bird alive in state $r=\left\{ {0,1} \right\}$ at age *a* at year *t* is not seen again alive or dead during the rest of the study:

$$\begin{aligned} \chi _{a,t} \left( 0\right)= & {} \left\{ {{ \begin{array}{ll} {1,} &{} {t=T} \\ {\left( {1-\lambda _{a,t}}\right) \left( {1-\phi _{a,t}}\right) +\phi _{a,t} \chi _{a+1,t+1} \left( 0\right) ,} &{} {t<T} \\ \end{array}}} \right. \\ \chi _{a,t} \left( 1\right)= & {} \left\{ {{ \begin{array}{ll} {1,} &{} {t=T} \\ {\left( {1-\lambda _{a,t}}\right) \left( {1-\phi _{a,t}}\right) +\phi _{a,t} \left\{ {\left( {1-F_{a,t}}\right) \chi _{a+1,t+1} \left( 0 \right) +F_{a,t} \left( {1-p_{a+1,t+1}}\right) \chi _{a+1,t+1} \left( 1 \right) } \right\} ,} &{} {t<T} \\ \end{array}}} \right. . \end{aligned}$$

The MRR dataset can be summarized using a set of sufficient statistics: (i) $n_{a,b,t} \left( {r,s}\right)$: number of birds observed in state $r=\left\{ {0,1} \right\}$ at age *a* in year *t* and next seen alive in state $s=\left\{ {0,1} \right\}$ aged $b+1$; (ii) $d_{a,b,t} \left( r\right)$: number of birds recovered dead at age *b* that were last observed alive in state $r=\left\{ {0,1}\right\}$ aged *a* in year *t*; and (iii) $v_{a,t} \left( r\right)$: number of birds seen alive (including initial release) for the last time in state $r=\left\{ {0,1} \right\}$ aged *a* in year *t*, and not recovered dead at a later encounter occasion.

Given that no murres are resighted in state 0, only the following terms are nonzero: $n_{a,b,t} \left( {1,1}\right) , d_{a,b,t} \left( 1\right)$ and $v_{a,t} \left( 1\right)$. The full age- and year-dependent likelihood of the MRR(C) dataset, taking into account restrictions in the relationships of the indices, is:

$$\begin{aligned} L\left( {{\varvec{n,d,v}}|{\varvec{\phi , \psi , p,\lambda }}}\right)= & {} \mathop {\prod }\limits _{a=1}^{A-1} \mathop {\prod }\limits _{b=a}^{A-1} \mathop {\prod }\limits _{t=a}^{\left( {T-1+a-b}\right) } \left\{ {O_{a,b,t} \left( {1,1}\right) ^{n_{a,b,t} \left( {1,1}\right) }\times D_{a,b,t} \left( 1\right) ^{d_{a,b,t} \left( 1\right) }} \right\} \\&\times \mathop {\prod }\limits _{a=1}^A \mathop {\prod }\limits _{t=a}^{T-1} \chi _{a,t} \left( 1 \right) ^{v_{a,t} \left( 1\right) }, \end{aligned}$$

which requires calculating the terms $$\chi _{a,t} \left( 0\right) , Q_{a,b,t} \left( {0,0}\right) , Q_{a,b,t} \left( {1,0}\right) $$ and $$Q_{a,b,t} \left( {1,1}\right) $$. Based on our previous analysis of this MRR dataset (Lahoz-Monfort et al. 2014), we simplify the above fully age- and time-dependent likelihood using the following age and year model structure (adult: defined as age $$a>5$$ years): (i) year-specific first-year survival parameters $$s_1 \left( t\right) $$ and adult survival $$s_a \left( t\right) $$; constant for $$2\mathrm{nd}$$ and $$3\mathrm{rd}$$-to-$$5\mathrm{th}$$ years of life (parameters $$s_2 $$ and $$s_{35} =s_3 =s_4 =s_5 )$$; (ii) year-specific resight probabilities, for three age classes ($$p_2 \left( t\right) , p_3 \left( t\right) $$ and $$p_{45} \left( t\right) $$ for 2, 3 and ‘4-to-5’ year olds) and adults $$p_a \left( t\right) $$, estimated independently for each banding area (A or B, indicated by superscript). We fix $$p_1 =0$$ as young murres do not return to their natal colony in their first year; (iii) we estimate fidelity in the two years before recruitment ($$F_5 $$ and $$F_6 )$$ but let $${F}_{1}={F}_{2}={F}_{3}={F}_{4}=1$$ for younger birds (uncommon recruitment at that early age) and $$F_a =1$$ for adults; (iv) a general trend of decreasing reporting probabilities has been noticed in several species in the UK (Robinson et al. 2009), so we fit a linear trend with time in $$\lambda _{a,t}$$ (on the logit scale) common to all ages: $$\alpha _0 +\alpha _1 y$$, with *y* the standardised years (from 1 to $$T-1)$$.

Some colour bands on immatures became worn and dropped off, so colour-band loss and recruitment into an area of low visibility are in principle confounded with emigration as individuals become unobservable alive but the stainless steel numbered bands may still be reported once the bird dies. These two processes can be separated from ‘true’ fidelity with the help of an IPM, as they impact very differently on population counts (Reynolds et al. 2009). We define the probability $\psi$ that an adult (marked as chick) retains a readable band and recruits (or continues breeding) at a visible location. Assuming $F_a =1$ and that $\psi$ only applies to birds that have started breeding (therefore adults), we can model the ‘retention of colour bands and recruitment to a visible location’ using the ‘fidelity’ parameter $\psi _{a,t} \left( {1,1}\right) =F_{a>6,t} =\psi$ for $a>6$.

For banding area A, let: $${\varvec{p_2^A}} =\left\{ {p_2^A \left( t \right) :t=3,\ldots , T} \right\} , {\varvec{p_3^A}} =\left\{ {p_3^A \left( t \right) :t=4,\ldots , T} \right\} , {\varvec{p_{45}^A}} =\left\{ {p_{45}^A \left( t\right) :t=5,\ldots , T} \right\} , {\varvec{p_a^A}} =\left\{ {p_a^A \left( t\right) :t=7,\ldots , T} \right\} $$; for area B: $${\varvec{p_2^B}} =\left\{ {p_2^B \left( t\right) :t=3,\ldots , 17} \right\} , {\varvec{p_3^B}} =\left\{ {p_3^B \left( t\right) :t=4,\ldots , 18} \right\} , {\varvec{p_{45}^B}} =\left\{ {p_{45}^B (t):t=5,\ldots , 20} \right\} , {\varvec{p_a^B}} =\left\{ {p_a^B \left( t\right) :t=7,\ldots , T} \right\} $$; and the complete parameter sets as $${\varvec{p_M^C}} =\big \{\varvec{p_2^A ,p_3^A, p_{45}^A}, \varvec{p_a^A, p_2^B, p_3^B, p_{45}^B, p_a^B} \big \}, {\varvec{F_M}} =\left\{ {F_5, F_6} \right\} $$, and immature survival $${\varvec{s_{iM}}} =\left\{ {{\varvec{s_1}}, s_2, s_{35}} \right\} $$. We treat areas A and B as two distinct datasets, $$\left\{ {{\varvec{n_A, d_A, v_A}}} \right\} $$ and $$\left\{ {{\varvec{n_B, d_B, v_B}}} \right\} $$, and construct the likelihoods for both areas, $$L_A^M $$ and $$L_B^M $$. The overall likelihood of the complete chick MRR(C) dataset can be constructed by multiplying both:

$$\begin{aligned}&L_{\mathrm{MRR}\left( \mathrm{C}\right) }^M \left( {{\varvec{n_M, d_M, v_M}} |{\varvec{s_{iM}, s_{aM}, p_M^C}}, \alpha _0, \alpha _1, {\varvec{F_M}}, \psi }\right) \\&\quad =L_A^M \left( {{\varvec{n_A, d_A, v_A}} |{\varvec{s_{iM}, s_{aM}, p^{A}}},\alpha _0, \alpha _1, {\varvec{F_M}}, \psi }\right) \\&\qquad \times L_B^M \left( {{\varvec{n_B, d_B, v_B}} |{\varvec{s_{iM}, s_{aM}, p^{B}}},\alpha _0, \alpha _1, {\varvec{F_M}}, \psi }\right) . \\ \end{aligned}$$

It is easier to handle this likelihood by realizing that it is product-multinomial (McCrea 2012). For releases aged *a* in year *t* in state 1, multinomial cell probabilities and corresponding observed cell numbers are $\big \{O_{a,a,t} ({1,1}),\ldots , O_{a,A,t} ({1,1}),D_{a,a,t} (1),\ldots , D_{a,A,t} (1),\chi _{a,t} (1) \big \}$ and $\left\{ {n_{a,a,t} \left( {1,1}\right) ,\ldots , n_{a,A,t} \left( {1,1}\right) ,d_{a,a,t} \left( 1\right) ,\ldots , d_{a,A,t} \left( 1\right) ,v_{a,t} \left( 1\right) } \right\} .$

### Breeding Population Counts: Population Model

In an IPM, population counts are modelled using a state-space population model (Buckland et al. 2004), which consists of two linked models. For each species S, the system process model describes the true population abundance $N_{xS} \left( {t+1}\right)$ for the different age classes *x* at year $t+1$ as a function of the previous year’s abundance. The structure of the population model for each species will have a degree of complexity (and realism) that depends on the datasets available and the ecology of the species. We specifically keep track of female abundance, which is sufficient to model the number of breeding pairs as our species are monogamous (Gaston and Jones 1998). A number $N_{aS} \left( t\right)$ of adult breeding females in year *t* will produce a single egg. Each egg has a probability $\rho _S \left( t\right)$ (overall productivity in year *t*) of hatching and the chick surviving until fledging, and a factor 0.5 takes into account that on average half of the chicks will be females (balanced sex ratio at fledging). Only a fraction of these fledglings will survive their first winter. The number of ‘age 1’ females at time $t+1$ can be modelled as a binomial distribution: $N_{1S} \left( {t+1}\right) \sim \hbox {bin}\left( {N_{aS} \left( t \right) ,\rho _S \left( t\right) s_{1S} \left( t\right) /2}\right)$, with $s_{1S} \left( t\right)$ the survival probability over the first year of life. The number of immature females of increasing age can be modelled in the same way using binomial distributions with corresponding age-specific survival.

We model recruitment using the median value of age at first breeding, denoted $$d_S $$ for species *S*. We use $$d_R =5$$ (median from Skokholm Island in Wales, $$n = 20$$; Lloyd and Perrins 1977), $$d_P =7$$ (median from the Isle of May, $$n = 108$$; Harris and Wanless 2011); and $$d_M =6$$ (median from the Isle of May, $$n = 42$$; Harris et al. 1994). Pre-breeders $$N_{d-1,S} \left( t\right) $$ represent the number of females in the year before first breeding. A non-negligible fraction of puffins and murres, and we assume razorbills, hatched at the Isle of May permanently emigrate and recruit to other colonies (Harris et al. 1996; Harris and Wanless 2011). We also assume that survival over the winter immediately before recruiting is equal to that of adult birds $$s_{aS} $$, hence the new recruits $$R_S \left( t\right) $$ to the female adult population in year *t* will be $$R_S \left( t\right) \sim \hbox {bin}\left( {N_{d-1,S} \left( {t-1}\right) , F_S s_{aS} \left( {t-1}\right) }\right) $$, where $$F_S $$ is pre-breeding fidelity.

In practice, we do not have enough data on immature razorbills and puffins to separate pre-breeder emigration from mortality, or to estimate age-dependent survival probabilities, so for these species we use a ‘combined survival’ parameter $$\phi _{cS} $$ which combines survival since fledging to the year before recruitment, and fidelity. We use letter $$\phi $$ following a common naming convention (White and Burnham 1999) to denote ‘apparent survival’ (where permanent emigration and mortality are confounded) instead of ‘true survival’ *s*. Razorbill and puffin new recruits can thus be modelled as $$R_S \left( t\right) \sim \hbox {bin}\left( {N_{aS} \left( {t-d_S}\right) , \rho _S \left( {t-d_S}\right) \phi _{cS} s_{aS} \left( {t-1}\right) /2}\right) .$$ From the adult population at time $$t-1$$, individuals will survive to year *t* with probability $$s_{aS} $$: $$S_S \left( t\right) \sim \hbox {bin}\left( {N_{aS} \left( {t-1}\right) , s_{aS} \left( {t-1}\right) }\right) $$. The total number of breeding females at year *t* will be the sum of surviving adults and new female recruits: $$N_{aS} \left( t\right) =S_S \left( t \right) +R_S \left( t\right) $$. Established breeding adults of the three species virtually never move to other colonies (Gaston and Jones 1998) so we assume no emigration ($$F_{aS} =1$$). A small pre-breeder immigration into the Isle of May population (Lloyd 1974; Halley and Harris 1993; Harris and Wanless 2011) occurs but our models assume no immigration due to lack of data to estimate it. Letting $$\rho _S \left( {t-d_S}\right) \frac{1}{2}\phi _{cS} =\tau _S \left( {t-d_S}\right) $$, the ‘likelihood’ of the system process model can be written as

$$\begin{aligned}&L_N^S \left( {{\varvec{R_S}}, {\varvec{S_S}} |\phi _{cS}, {\varvec{s_{aS}}}, {\varvec{\rho _S}}}\right) \\&\quad =\mathop {\prod }\limits _{t=d+1}^T \left[ {\left( {{ \begin{array}{l} {N_{aS} \left( {t-d_S}\right) } \\ {R_S \left( t\right) } \\ \end{array}}}\right) \left\{ {\tau _S \left( {t-d_S}\right) s_{aS} \left( {t-1}\right) } \right\} ^{R_S \left( t\right) }} \right. \\&\qquad \times \left\{ {1-\tau _S \left( {t-d_S}\right) s_{aS} \left( {t-1}\right) } \right\} ^{N_{aS} \left( {t-d_S}\right) -R_S \left( t\right) } \\&\qquad \left. \times \left( {{ \begin{array}{l} {N_{aS} \left( {t-1}\right) } \\ {S_S \left( t\right) } \\ \end{array}}}\right) \left\{ {s_{aS} \left( {t-1}\right) } \right\} ^{S_S \left( t\right) }\left\{ {1-s_{aS} \left( {t-1}\right) } \right\} ^{N_{aS} \left( {t-1}\right) -S_S \left( t\right) } \right] . \end{aligned}$$

$$L_N^S $$ is not a true likelihood strictly speaking (it does not involve the observed data) but rather a description of the unobserved underlying population changes.

For murres, we have direct information regarding immature survival (MRR(C) dataset) so we incorporate immature survival and fidelity parameters defined in Sect. 2.4 into the population model. New recruits ${\varvec{R_M}} =\left\{ {R_M \left( t\right) :t=7,\ldots , T} \right\}$, surviving adult females ${\varvec{S_M}} =\left\{ {S_M \left( t \right) :t=7,\ldots , T} \right\}$ and adult breeding females ${\varvec{N_{aM}}} =\left\{ {N_{aM} \left( t\right) : t=7,\ldots , T} \right\}$ can be modelled as

$$\begin{aligned}&R_M \left( t\right) \sim \hbox {bin}\left( {N_{aM} \left( {t-6}\right) , B\left( {t-6}\right) \rho _M \left( {t-6}\right) \frac{1}{2}s_1 \left( {t-6}\right) s_2 s_{35}^3 F_5 F_6 s_{aM} \left( {t-1}\right) }\right) ,\\&S_M \left( t\right) \sim \hbox {bin}\left( {N_{aM} \left( {t-1}\right) , s_{aM} \left( {t-1}\right) }\right) ,\\&N_{aM} \left( t\right) =R_M \left( t\right) +S_M \left( t\right) . \end{aligned}$$

Letting $B\left( {t-6}\right) \rho _M \left( {t-6}\right) \frac{1}{2}s_1 \left( {t-6}\right) s_2 s_{35}^3 F_5 F_6 =\tau _M \left( {t-6}\right)$, the system process model is

$$\begin{aligned}&L_N^M \left( {{\varvec{R_M, S_M}} |{\varvec{s_M, F_M, s_{aM}, \rho _M}}}\right) \\&\quad =\mathop {\prod }\limits _{t=7}^T \left[ {\left( {{ \begin{array}{l} {N_{aM} \left( {t-6}\right) } \\ {R_M \left( t\right) } \\ \end{array}}}\right) \left\{ {\tau _M \left( {t-6}\right) s_{aM} \left( {t-1}\right) } \right\} ^{R_M \left( t\right) }} \right. \\&\qquad \times \left\{ {1-\tau _M \left( {t-6}\right) s_{aM} \left( {t-1}\right) } \right\} ^{N_{aM} \left( {t-6}\right) -R_M \left( t\right) } \\&\qquad \left. {\times \left( {{ \begin{array}{l} {N_{aM} \left( {t-1}\right) } \\ {S_M \left( t\right) } \\ \end{array}}}\right) \left\{ {s_{aM} \left( {t-1}\right) } \right\} ^{S_M \left( t\right) }\left\{ {1-s_{aM} \left( {t-1}\right) } \right\} ^{N_{aM} \left( {t-1}\right) -S_M \left( t\right) }} \right] . \end{aligned}$$

### Breeding Population Counts: Observation Model

An observation model relates an imperfect observation of abundance (counts) to the true state of the system: the true abundance of breeding females $$N_{aS} \left( t\right) $$. Island-wide population counts $$x_S \left( t\right) $$ of adult breeding pairs (and hence females) have been conducted annually for murres and razorbills and less frequently for puffins. We model these counts with a normally distributed observation error $$x_S \left( t\right) \sim \hbox {N}\left( {N_{aS} \left( t\right) ,\sigma _{xS}^2}\right) $$, for $$t=d_s +1,{\ldots },T$$. This assumes that false negatives are approximately as likely as false positives. Counts for the first $$d_S $$ years cannot be related to $$N_{aS}$$ abundance modelled as a function of parameters and immature abundance since there is no direct source of information about the abundance of younger age classes for the first $$d_S $$ years. We use these to initialize the population model for that period by setting informative normal priors for the adult population, assuming same variance as for observation error: $$N_{aS} \left( t\right) \sim \hbox {N}\left( {x_S \left( t\right) , \sigma _{xS}^2}\right) $$, for $$t=1,{\ldots },d_s$$. Letting $${\varvec{x_S}} =\left\{ {{{x_S}} \left( t\right) :t=d_S +1,\ldots , T} \right\} $$, the observation process likelihood is

$$\begin{aligned} L_{\mathrm{OBS}}^S \left( {{\varvec{x_S}} |{\varvec{N}}_{aS}, \sigma _{xS}^2}\right) =\mathop {\prod }\limits _{t=d+1}^T \left[ {\frac{1}{\sigma _{xS} \sqrt{2\pi }}\cdot \exp \left( {-\frac{\left\{ {x_S \left( t\right) -N_{aS} \left( t \right) } \right\} ^{2}}{2\sigma _{xS}^2}}\right) } \right] . \end{aligned}$$

Puffin counts are only available for 7 non-consecutive years: 1984 and 1989 are used as initialization priors (with missing years interpolated linearly) and the model is fitted to counts from 1992, 1998, 2003, 2008 and 2009, but is able to estimate adult population for all years.

Finally, the likelihood of the state-space population model for each species $S\,(L_{\mathrm{POP}}^{S})$ is the product of the likelihood of the observation model $(L_{\mathrm{OBS}}^{S})$ and the system process model $(L_{N}^{S})$. This represents the complete-data likelihood, which includes the unobserved data (true population abundances). This expression is not easily evaluated (e.g. in frequentist inference); we circumvent this limitation using Bayesian inference, as explained in Sect. 4.

### Joint Likelihood: ssIPMs

Assuming independence between the different datasets involved, the joint likelihood of each single-species IPM can be found by multiplying the likelihoods of the different components:

$$\begin{aligned}&L_{\mathrm{IPM}}^S \left( {{\varvec{x_S, m_S, P_S}} |{\varvec{R_S, S_S}}, \phi _{cS}, {\varvec{s_{aS}}}, {\varvec{p}}_{\varvec{S}}^*, a_S, {\varvec{\rho _S}}}\right) \\&\quad =L_{\mathrm{POP}}^S \left( {{\varvec{x_S}} |{\varvec{R}}_S, {\varvec{S_S}}, \phi _{cS}, {\varvec{s_{aS}}}, {\varvec{\rho _S}}, \sigma _{xS}^2}\right) \times L_{\mathrm{MR}\left( \mathrm{A}\right) }^S \left( {{\varvec{m_S}} |{\varvec{s_{aS}}}, {\varvec{p}}_{\varvec{S}}^*, a_S}\right) \times L_{\mathrm{BS}}^S \left( {{\varvec{P_S}} |{\varvec{\rho _S}}}\right) . \end{aligned}$$

For murres, this joint likelihood contains also the components related to the extra datasets:

$$\begin{aligned}&L_{\mathrm{IPM}}^M \left( {{\varvec{x_M, m_M, n_M, d_M, v_M, P_M, \xi _M}} |{\varvec{N_M, s_{aM}, \rho _M}}, \sigma _{xM}^2, {\varvec{p}}_{\varvec{M}}^*, a_{\mathrm{M}}, {\varvec{s_M, p_M^C}}, \alpha _0, \alpha _1, {\varvec{F_M}}, \psi , {\varvec{B_M}}}\right) \\&\quad =L_{\mathrm{POP}}^M \left( {{\varvec{x_M}} |{\varvec{R_M, S_M, s_M, F_M, s_{aM}, \rho _M}}, \sigma _{xM}^2}\right) \times L_{\mathrm{MR}\left( \mathrm{A}\right) }^M \left( {{\varvec{m_M}} |{\varvec{s_{aM}}}, {\varvec{p}}_{\varvec{M}}^*, a_M}\right) \\&\qquad \times L_{\mathrm{MRR}\left( \mathrm{C}\right) }^M \left( {{\varvec{n_M, d_M, v_M}} |{\varvec{s_{iM}, s_{aM}, p_M^C}}, \alpha _0, \alpha _1, {\varvec{F_M}}, \psi }\right) \times L_{\mathrm{BS}}^M \left( {{\varvec{P_M}} |{\varvec{\rho _M}}}\right) \times L_{\mathrm{NB}}^M \left( {{\varvec{\xi _M}} |{\varvec{B_M}}}\right) . \end{aligned}$$

We use different adult resight probabilities for murres marked as chicks (${\varvec{p_M^C}})$ than for those marked as adults (${\varvec{p_M}}$, derived from ${\varvec{p}}_{\varvec{M}}^*$ and ${\varvec{a_M}})$, as high resight probabilities are expected for the latter (more resight effort and highly likely to return to the same breeding spot where banded). Adult survival ${\varvec{s_{aM}}}$ is a common parameter for the adult MR likelihood $L_{MR\left( A\right) }^M$ and the chick MRR likelihood $L_{\mathrm{MRR}\left( \mathrm{C}\right) }^M$. Table 1 summarizes the shared parameters.

**Table 1:** List of parameters involved in the msIPM, specifying in which model component for which species they appear.

Parameters	$\hbox {BS}_{R}$	$\hbox {MR(A)}_{R}$	$\hbox {POP}_{R}$	$\hbox {BS}_{P}$	$\hbox {MR(A)}_{P}$	$\hbox {POP}_{P}$	$\hbox {BS}_{M}$	$\hbox {NB}_{M}$	$\hbox {MR(A)}_{M}$	$\hbox {MRR(C)}_{ M}$	$\hbox {POP}_{M}$
${\varvec{\delta _\rho }}, \sigma _{\delta \rho }^2$	$\checkmark$		$\checkmark$	$\checkmark$		$\checkmark$	$\checkmark$				$\checkmark$
${\varvec{\delta _\phi }}, \sigma _{\delta \phi }^2$		$\checkmark$	$\checkmark$		$\checkmark$	$\checkmark$			$\checkmark$	$\checkmark$	$\checkmark$
[S] $\beta _{\rho R}, {\varvec{\varepsilon _{\rho R}}}, \sigma _{\varepsilon \rho R}^2$	$\checkmark$		$\checkmark$								
[S] $\beta _{\phi R}, {\varvec{\varepsilon _{\phi R}}}, \sigma _{\varepsilon \phi R}^2$		$\checkmark$	$\checkmark$								
${\varvec{\rho _R}}$	$\checkmark$		$\checkmark$								
${\varvec{s_{aR}}}, {\varvec{p}}_{\varvec{R}}^*, a_R, {\varvec{p_R}}, \overline{{\varvec{p}}}_{\varvec{R}}$		$\checkmark$	$\checkmark$								
$\phi _{cR}, \sigma _{xR}^2, R_R, S_R, N_R$			$\checkmark$								
[S] $\beta _{\rho P}, {\varvec{\varepsilon _{\rho P}}}, \sigma _{\varepsilon \rho P}^2$				$\checkmark$		$\checkmark$					
[S] $\beta _{\phi P}, {\varvec{\varepsilon _{\phi P}}}, \sigma _{\varepsilon \phi P}^2$					$\checkmark$	$\checkmark$					
${\varvec{\rho _P}}$				$\checkmark$		$\checkmark$					
${\varvec{s_{aP}}}, {\varvec{p}}_{\varvec{P}}^*, a_P, {\varvec{p_P}}, \overline{{\varvec{p}}}_{\varvec{P}}$					$\checkmark$	$\checkmark$					
$\phi _{cP}, \sigma _{xP}^2, R_P, S_P, N_P$						$\checkmark$					
[S] $\beta _{\rho M}, {\varvec{\varepsilon _{\rho M}}}, {{\sigma }}_{{{\varepsilon \rho M}}}^2$							$\checkmark$				$\checkmark$
[S] $\beta _{\phi M}, {\varvec{\varepsilon _{\phi M}}}, {{\sigma }}_{{{\varepsilon \phi M}}}^2$									$\checkmark$	$\checkmark$	$\checkmark$
${\varvec{\rho _M}}$							$\checkmark$				$\checkmark$
${\varvec{B}}$								$\checkmark$			$\checkmark$
${\varvec{s_{aM}}}, {\varvec{p}}_{\varvec{M}}^*, a_M, {\varvec{p_M}}, \overline{{\varvec{p}}}_{\varvec{M}}$									$\checkmark$	$\checkmark$	$\checkmark$
${\varvec{s_1}}, s_2, s_{35}, F_5, F_6, \psi , {\varvec{r}},\alpha _0, \alpha _1$										$\checkmark$	$\checkmark$
${\varvec{p_2^A, p_3^A, p_{45}^A, p_a^A, p_2^B, p_3^B, p_{45}^B, p_a^B}}$										$\checkmark$	$\checkmark$
${\phi }_{cM}, {\sigma }_{xM}^2, R_M, S_M, N_M$											$\checkmark$

Column names refer to likelihood components names described in the ssIPM sections. ‘*R*’, ‘*P*’ and ‘*M*’ refer to razorbill, puffin and murre, respectively. BS = Breeding Success; MR(A) = Adult Mark-Resight; POP = Population; NB = Non-Breeding; MRR(C) = Chick Mark-Resight-Recovery. Year-specific parameters shown in bold. Synchrony-related parameters marked with ‘[S]’; first 2 rows show parameters related to all species.

## Multi-Species Integrated Population Model (msIPM)

The ssIPMs bring together datasets and model components that relate to each species independently. We now model jointly the ssIPMs for the three alcids in a single multi-species integrated model (msIPM) by applying a recently developed multi-species synchrony framework to study the degree of common year-to-year variation in demographic parameters. We have already conducted synchrony analyses independently for survival (Lahoz-Monfort et al. 2011) and productivity (Lahoz-Monfort et al. 2013); we do so here within the context of integrated population modelling. We briefly describe the synchrony components of the model here and refer to the references above for details; we note also some novel methodological developments in the estimation of multi-species synchrony in relation to covariates (Swallow et al. 2016). For adult survival and productivity independently, we add year random effects on the logistic scale, either common to all species ($\delta _\phi \left( t\right) , \delta _\rho \left( t\right)$, representing the community common response) or species-specific ($\varepsilon _{\phi S} \left( t\right) , \varepsilon _{\rho S} \left( t \right) )$, with subscripts $\phi$ and $\rho$ denoting adult survival and productivity:

$$\begin{aligned} \hbox {logit}\left( {\rho _S \left( t\right) }\right)= & {} \beta _{\rho S} +\delta _\rho \left( t\right) +\varepsilon _{\rho S} \left( t\right) ,\quad t=1,\ldots , T, S=1,2,3, \\ \hbox {logit}\left( {s_{aS} \left( t \right) }\right)= & {} \beta _{\phi S} +\delta _\phi \left( t \right) +\varepsilon _{\phi S} \left( t\right) , \quad t=1,\ldots ,T, S=1,2,3. \end{aligned}$$

The normally distributed random effects are considered independent (across years and species), as: $\delta _\phi \left( t\right) \sim N\left( {0,\sigma _{\delta \phi }^2}\right) , \varepsilon _{\phi S} \left( t\right) \sim N\left( {0,\sigma _{\varepsilon \phi S}^2}\right) , \delta _\rho \left( t\right) \sim N\left( {0,\sigma _{\delta \rho }^2}\right) , \varepsilon _{\rho S} \left( t\right) \sim N\left( {0,\sigma _{\varepsilon \rho S}^2}\right)$, where $t=1,{\ldots },T$ for productivity and $t=1,{\ldots },T-1$ for survival, and $S=1,2,3$. For our three species, the likelihoods corresponding to the adult MR and breeding success datasets, as well as the population model, now depend on the random terms and intercepts $\beta _{\rho s}$ and $\beta _{\phi s}$. For instance, for razorbill breeding success data we have $L_{\mathrm{BS}}^R \left( {{\varvec{P_R}} |{\varvec{\delta _\rho }}, {\varvec{\varepsilon _{\rho R}}}, \sigma _{\delta \rho }^2, \sigma _{\varepsilon \rho R}^2, \beta _{\rho s}}\right) =f_{\mathrm{BS}}^R \left( {{\varvec{P_R}} |{\varvec{\delta _\rho , \varepsilon _{\rho R}}}, \beta _{\rho s}}\right) f_{\delta \rho } \left( {{\varvec{\delta _\rho }} |\sigma _{\delta \rho }^2}\right) f_{\varepsilon \rho }^R \left( {{\varvec{\varepsilon _{\rho R}}} |\sigma _{\varepsilon \rho R}^2 }\right) ,$ where $f\left( .\right)$ denotes pdf or pmf (for continuous or discrete data, respectively). The part of the msIPM likelihood that corresponds to the complete razorbill dataset is a joint distribution over the model parameters, the random terms and the unobserved population abundance

$$\begin{aligned}&f_{\mathrm{IPM}}^R \left( {{\varvec{x_R, m_R, P_R}} |{\varvec{R}}_{{R}}, {\varvec{S_R}}, \phi _{cR}, {\varvec{\delta _\phi , \varepsilon _{\phi R}}}, \sigma _{\delta \phi }^2, \sigma _{\varepsilon \phi R}^2, \beta _{\phi R}, {\varvec{p}}_{\varvec{R}}^*, a_R, {\varvec{\delta _\rho }}, {\varvec{\varepsilon _{\rho R}}}, \sigma _{\delta \rho }^2, \sigma _{\varepsilon \rho R}^2, \beta _{\rho R}}\right) \\&\quad =f_{\mathrm{POP}}^R \left( {{\varvec{x_R}}|{\varvec{R}}_R, {\varvec{S_R}}, \phi _{cR}, {\varvec{\delta _\phi , \varepsilon _{\phi R}}}, \beta _{\phi R}, {\varvec{\delta _\rho , \varepsilon _{\rho R}}}, \beta _{\rho R}, \sigma _{xR}^2}\right) \\&\qquad \times f_{\mathrm{MR}\left( \mathrm{A}\right) }^R \left( {{\varvec{m_R}} |{\varvec{\delta _\phi , \varepsilon _{\phi R}}}, \beta _{\phi R}, {\varvec{p}}_{\varvec{R}}^*, a_R}\right) f_{\mathrm{BS}}^R \left( {{\varvec{P_R}} |{\varvec{\delta _\rho , \varepsilon _{\rho R}}}, \beta _{\rho R}}\right) \\&\qquad \times f_{\delta \phi } \left( {{\varvec{\delta _\phi }} |\sigma _{\delta \phi }^2}\right) f_{\varepsilon \phi }^R \left( {{\varvec{\varepsilon _{\phi R}}} |\sigma _{\varepsilon \phi R}^2}\right) f_{\delta \rho } \left( {{\varvec{\delta _\rho }} |\sigma _{\delta \rho }^2}\right) f_{\varepsilon \rho }^R \left( {{\varvec{\varepsilon _{\rho R}}} |\sigma _{\varepsilon \rho R}^2}\right) \end{aligned}$$

The likelihood components for puffin and murre datasets can be written similarly. Denoting the complete dataset ${\varvec{h_S}}$ and all species-specific parameters and auxiliary variables ${\varvec{\theta _S}}$, the msIPM joint likelihood can be written as a function of each species’ IPM distribution conditional on the random terms, and the pdf of the random terms:

$$\begin{aligned}&L_{\mathrm{msIPM}} \left( {{\varvec{h_R, h_P, h_M}} |{\varvec{\theta _R, \theta _P, \theta _M, \delta _\phi }}, \sigma _{\delta \phi }^2, {\varvec{\delta _\rho }}, \sigma _{\delta \rho }^2}\right) \\&\quad =f_{\mathrm{IPM}}^R \left( {{\varvec{h_R}} |{\varvec{\theta _R, \delta _\phi , \delta _\rho }}}\right) \times f_{\mathrm{IPM}}^P \left( {{\varvec{h_P}} |{\varvec{\theta _P, \delta _\phi , \delta _\rho }}}\right) \\&\qquad \times f_{\mathrm{IPM}}^M \left( {{\varvec{h_M}} |{\varvec{\theta _M, \delta _\phi , \delta _\rho }}}\right) \times f_{\delta \phi } \left( {{\varvec{\delta _\phi }} |\sigma _{\delta \phi }^2}\right) f_{\delta \rho } \left( {{\varvec{\delta _\rho }} |\sigma _{\delta \rho }^2}\right) . \end{aligned}$$

The indices of synchrony for each species *S* can be derived from the random effects variances as $I_{\phi S} =\frac{\hat{\sigma }_{\delta \phi }^2}{\hat{\sigma }_{\delta \phi }^2 +\hat{\sigma }_{\varepsilon \phi S}^2}$ and $I_{\rho S} =\frac{\hat{\sigma }_{\delta \rho }^2}{\hat{\sigma }_{\delta \rho }^2 +\hat{\sigma }_{\varepsilon \rho S}^2}$. They represent the synchrony of species S with the rest of the species: the amount of between-year variance for species *S* that is common to all the others (common random terms $\delta (t))$. High values for a species indicate that most of its year-to-year variation is synchronous to the set. ‘Community-level’ synchrony parameters (common random terms and their variances) are shared across species, rendering the model multi-species. Table 1 lists the estimated parameters for the three species, and where they appear in the likelihood.

## Bayesian Analysis

To carry out Bayesian inference, the joint posterior density is constructed as the product of the likelihood and the prior densities for the parameters involved in the model

$$\begin{aligned}&\pi _{\mathrm{msIPM}} \left( {{\varvec{\theta _R, \theta _P, \theta _M, \delta _\phi }}, \sigma _{\delta \phi }^2, {\varvec{\delta _\rho }}, \sigma _{\delta \rho }^2 |{\varvec{h_R, h_P, h_M}}}\right) \\&\quad \propto L_{\mathrm{msIPM}} \left( {{\varvec{h_R, h_P, h_M}} |{\varvec{\theta _R, \theta _P, \theta _M, \delta _\phi }}, \sigma _{\delta \phi }^2, {\varvec{\delta _\rho }}, \sigma _{\delta \rho }^2}\right) \\&\quad \times \pi \left( {{\varvec{\theta _R, \theta _P, \theta _M, \delta _\phi }}, \sigma _{\delta \phi }^2, {\varvec{\delta _\rho }}, \sigma _{\delta \rho }^2}\right) . \end{aligned}$$

Random effects and the unobserved population abundances are treated as auxiliary variables, with MCMC chains updated at each step. The MCMC algorithm samples from the joint posterior distribution, averaging out the auxiliary variables and obtaining samples from the marginal posterior distributions for all parameters of interest. We specify priors to be as uninformative as possible: (i) flat proper priors $${U}\left( {0,1}\right) $$ for probabilities $$\phi _{\mathrm{cS}}, F_5, F_6, \psi , {\varvec{B}}, {\varvec{p^{A},p^{B}}}, {\varvec{s_1}}, s_2, s_{35} $$; (ii) flat proper priors $${U}\left( {-5,5}\right) $$ for intercepts of logistic regressions ($$\beta _{\phi s}, \beta _{\rho s} )$$, trap dependence $$a_s $$, band-recovery probability parameters $$\alpha _0, \alpha _1 $$; (iii) normally distributed low-information priors $$N\left( {0,10^{4}}\right) $$ for resight probabilities $${\varvec{p}}_{\varvec{s}}^*$$ (in the logit scale); (iv) flat proper priors $${U}\left( {0,3}\right) $$ for SDs of random terms ($$\sigma _{\delta \phi }, \sigma _{\delta \rho } ,\sigma _{\varepsilon \phi s}, \sigma _{\varepsilon \rho s} )$$, following Gelman (2006); and (v) flat proper priors for SD $$\sigma _{xs} $$ of the observation errors: $${U}\left( {0,15000}\right) $$ for puffins, $${U}\left( {0,5000}\right) $$ for the other species. We check after analysis that marginal posteriors are not limited by the intervals of uniform priors.

All analyses are conducted with program JAGS v2.2.0 (Plummer 2003), assessing convergence of chains with the $\hat{R}$ Gelman–Rubin diagnostic (Gelman and Rubin 1992) calculated in R package CODA (Plummer et al. 2006) for all parameters from two chains started at different values. The statistic shows no evidence of lack of convergence after one million MCMC iterations ($\hat{R} \le 1.03$, for most parameters $<1.02$; the only $\hat{R} > 1.05$ is found for $p_G^*$ for the last year (2009), which is estimated very imprecisely even in a MR-only analysis, and which has little impact on the population model). To explore the effect of multi-species integration in the estimation of demographic parameters, we also fit the three single-species IPMs (as described in Section 2 without synchrony structure), using program JAGS as above.

**Fig. 1:**
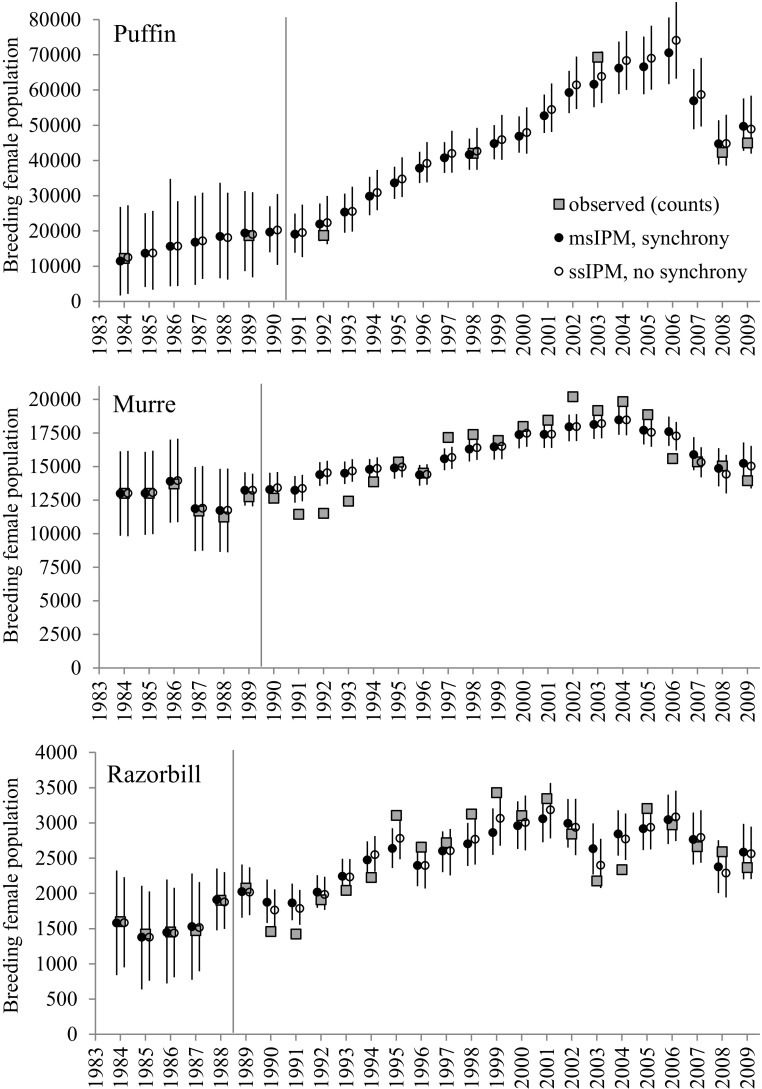
Comparison of female population abundance (median and symmetric 95% CIs) for puffin, murre and razorbill estimated from multi-species IPMs; *solid black circles*) and single-species IPMs (*open black circles*), and complete island-wide counts (*grey squares*). *Vertical lines* mark the end of the initialization period for the population models.

## Results

### Multi-species IPM

We obtain one million MCMC samples (thinned to 1/40th to reduce memory requirements) after a burn-in of one million samples (5.9 days on a 3.4 GHz processor). Marginal posteriors are obtained using MCMC for 1031 model parameters and summarized by the median and symmetric 95% Credible Intervals. Of these, 390 are derived deterministically from others.

Figure 1 shows the estimated true adult female population abundance for the period 1984–2009 for the three species. Puffins show the greatest change, strongly increasing from 11,390 in 1984 to 70,540 in 2006, followed by an unprecedented population crash (37%) to 44,710 pairs two years later. Despite population counts only being available for five years after 1990, the IPM is able to fit well the initial steady increase and estimates the population peak as taking place in 2006. Murre abundance shows a similar pattern but with less variation: steady increase for most of the period (from $\sim$13,000 to 18,450 in 2004) followed by a substantial decline (19%). Estimates fit the counts reasonably well, save for some discrepancy in 1991–1993 and 2002; despite its more flexible structure, some assumptions are still made (e.g. constant immature survival over their first winter). Razorbill numbers are substantially smaller but follow a similar pattern of steady increase (from $\sim$1500 to 3045 in 2006) followed by a drop (22%) in 2008; another population drop is apparent in 2003. Estimates fit the general pattern of counts well, but with greater annual variation: model structure may be slightly rigid (e.g. constant combined immature survival $\phi _{cR}$ imposed by lack of data on immature survival); attempts to fit more flexible models (e.g. year-specific $\phi _{cR} \left( t \right) )$ gave very imprecise estimates of $\phi _{cR}$. The model captures the general population trend, in agreement with the demographic variation.

**Fig. 2:**
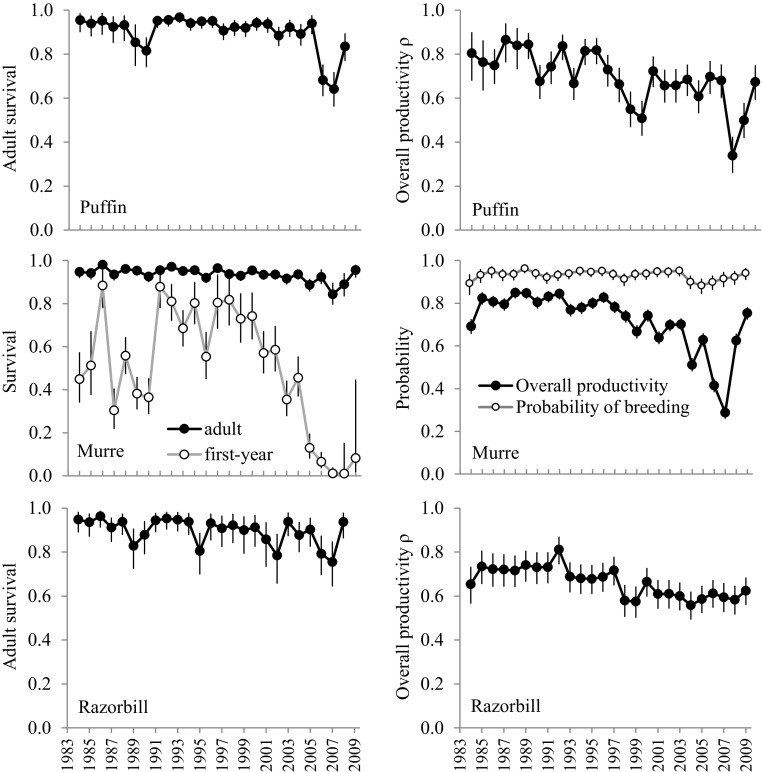
Estimates (median and symmetric 95% CI) of adult survival ${\varvec{s_{as}}} \left( {\varvec{t}}\right)$ and productivity ${\varvec{\rho _s}} \left( {\varvec{t}} \right)$ for puffin, murre and razorbill; and first-year survival ${\varvec{s_1}} \left( {\varvec{t}}\right)$ and probability of breeding ${\varvec{B}}\left( {\varvec{t}}\right)$ for murre.

Estimation of adult survival and productivity (Fig. 2) and related synchrony indices (Table 2) is in line with that of analyses of each demographic parameter separately (Lahoz-Monfort et al. 2011, 2013); we refer the reader to these for an ecological interpretation of high/low indices. The chick MRR murre dataset provides much information on immature survival. Estimates of $1\mathrm{st}$-year survival show very high yearly variability (from 0.9 to almost 0; arithmetic mean $=$ 0.502), with a steady decline from late 1990s reaching extremely low levels in 2004–2008 (Fig. 2), again in line with a previous ssIPM (Lahoz-Monfort et al. 2014). Survival estimates for older age classes increase with age towards the typically high values of adults (arithmetic mean for $\hat{s}_{aM} \left( t \right) : 0.936$), with $\hat{s}_2 = 0.763\, (0.717, 0.809), \hat{s}_{35} = 0.898 (0.876, 0.920)$. We estimate $<20\%$ of immature murres permanently emigrate to other colonies before recruitment: $\hat{F}_5 =0.865\, (0.820, 0.912)$ and $\hat{F}_6 =0.834\,(0.786, 0.884)$. For murres banded as chicks, once recruited into this population, the probability of keeping a readable band and breeding at a visible location is $\hat{\psi } =0.850\, (0.832,0.868)$; i.e. becoming non-resightable at a rate of 15%/year. Estimated combined juvenile survival is $\hat{\phi }_{cP} =0.761\,(0.621, 0.905)$ and $\hat{\phi }_{cR} =0.501\,(0.402, 0.614)$.

### Multi-species: Synchrony and Shrinkage of the Estimates

**Fig. 3:**
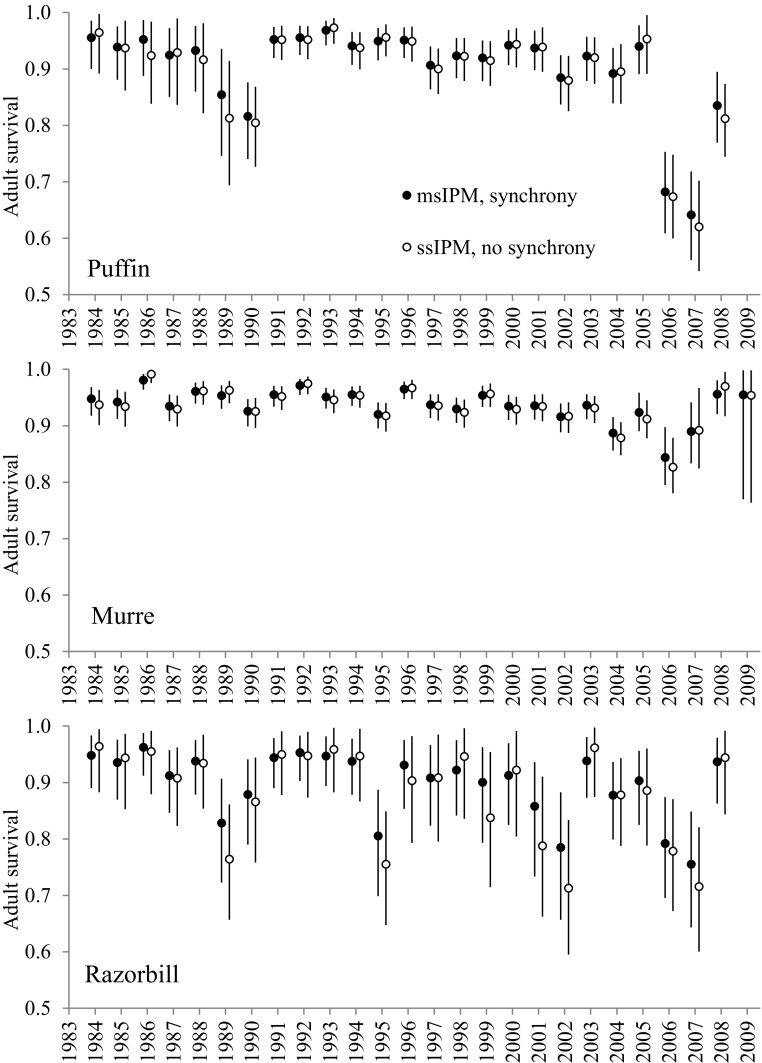
Comparison of adult survival (median and symmetric 95% CIs) for puffin, murre and razorbill estimated from msIPMs (*solid black circles*) and ssIPMs (*open black circles*).

The estimation of synchrony in adult survival and productivity uses random effects, which are known to produce an effect of ‘shrinkage towards the mean’ (Schaub and Kéry 2012). We find little shrinkage for murre adult survival (Fig. 3), given that the species contributes most MR data (1.5 and 5 times more banded murres than puffins and razorbills, respectively). Shrinkage is visible in puffins (particularly 1986, 1989 and 2008) and prevalent in razorbills (e.g. 1989 and 1995), the two species with least data. We note a similar effect on productivity estimates (not shown), as there are over five times more monitored murres than the two other species. Shrinkage in demographic parameters does not appear to have a strong effect on our estimation of population abundance (Fig. 1), causing only a slight smoothing in the most extreme years for species with fewer data (e.g. 1995 and 2003 for razorbills; 2006 for puffins).

**Table 2:** Estimates (median and 95% CI in brackets) of the msIPM constant parameters for puffin, murre and razorbill.

	Puffin	Murre	Razorbill
$\hat{\sigma }_{xs}$	5551 (2501, 11,920)	1503 (1055, 2284)	358 (237, 550)
Trap-dep. $\hat{a}_s$	1.928 (1.633, 2.227)	3.240 (2.840, 3.618)	1.826 (1.234, 2.428)
Intercept $\hat{\beta }_{\phi s}$	2.436 (2.119, 2.823)	2.789 (2.548, 3.056)	2.319 (1.976, 2.721)
$\hat{\sigma }_{\varepsilon \phi s}$	0.625 (0.391, 0.970)	0.261 (0.019, 0.597)	0.519 (0.101, 0.961)
$\hat{\sigma }_{\delta \phi }$	0.493 (0.272, 0.748)		
$\hat{I}_{\phi s}$	0.383 (0.108, 0.697)	0.787 (0.270, 0.999)	0.477 (0.122, 0.968)
Intercept $\hat{\beta }_{\rho s}$	0.890 (0.627, 1.159)	1.002 (0.751, 1.250)	0.689 (0.518, 0.870)
$\hat{\sigma }_{\varepsilon \rho s}$	0.498 (0.300, 0.762)	0.491 (0.344, 0.702)	0.109 (0.006, 0.337)
$\hat{\sigma }_{\delta \rho }$	0.357 (0.237, 0.540)		
$\hat{I}_{\rho s}$	0.340 (0.127, 0.690)	0.344 (0.138, 0.651)	0.913 (0.545, 1.000)
$\hat{\alpha }_0$	NA	−3.084 (−3.225, −2.949)	NA
$\hat{\alpha }_1$	NA	−0.687 (−0.837, −0.540)	NA
$\hat{\phi }_c$	0.761 (0.621, 0.905)	NA	0.501 (0.402, 0.614)

*NA* not applicable for the species.

## Discussion

Combining information from demography and abundance in integrated population modelling is a relatively recent but promising development in the area of statistical modelling of wildlife populations (Besbeas et al. 2002; Péron and Koons 2012). In this paper, we construct IPMs independently for 3 alcid species that breed at a single place by combining demographic datasets available with island-wide population counts; these include the first IPMs produced for puffins and razorbills. We move a step further by integrating data also across species in what is to our knowledge the first multi-species synchrony IPM. The analyses show dramatic changes in the population of alcids at the Isle of May, as well as strong fluctuations in the underlying demographic parameters, whose relation to abundance is modelled explicitly through the IPMs. The indices of synchrony indicate that, for adult survival and productivity, the common year-to-year variation represents only a medium-to-small part of the overall fluctuations. The low productivity synchrony in puffins and murres is driven by the rather constant (but slightly declining) razorbill productivity. Both puffins and murres had very poor breeding seasons around 2007 while razorbill productivity was not affected; all three share a long-term decline in productivity. msIPMs should provide a more robust framework for estimating synchrony, particularly when some species have fewer data and the population model helps estimate demographic parameters; this was not evident in our case due to our rich datasets.

Depending on data availability and model structure, demographic integration can sometimes permit estimation of parameters which cannot be estimated from independent analyses of the individual datasets involved (Besbeas et al. 2002). Our IPMs allow the estimation of combined juvenile survival for razorbills and puffins, two species without direct data about the fate of individual juvenile birds (a common situation for long-lived, pelagic species). IPMs also improve the estimation of abundance, compared to naïve counts with observation error. Despite the apparent complexity of the msIPM (641 non-derived parameters), the amount of data is correspondingly very large (e.g. 17,303 resightings for the three species combined), as it is the result of combining eight datasets, an important investment in field effort over this period of time.

IPMs can also separate true population abundance and observation error. Using counts without allowing for observation error may dilute the relationship of abundance to other processes of interest (e.g. density-dependence, Freckleton et al. 2006). IPMs also allow the estimation of abundance in years when counts are not available. In our study, this enabled us to identify when the puffin population crashed on the Isle of May. While population models can be populated with independent estimates of demographic parameters, their joint estimation within an IPM ensures that demography ‘agrees’ with an (imperfect) observation of the variation of population abundance (counts). Associated with species integration in the msIPM (and synchrony estimation) is some degree of shrinkage towards the community mean, which in our case was only slight for adult survival and even smaller for productivity; this is also associated with increases in precision. As expected, the effect is stronger for the species that contributes the least data.

IPMs commonly have many parameters. Methods and guidelines for checking potential identifiability issues and overall goodness-of-fit of IPMs are still under development, but exploring each separate sub-model may provide informative detail about the origin of a potential lack of fit in the overall model (Besbeas and Morgan 2014). In our case, trap dependence in resight probabilities was already introduced in the model following an assessment of the MR components (Lahoz-Monfort et al. 2011) and the more complex murre chick MRR model was scrutinized in a previous analysis (Lahoz-Monfort et al. 2014). Model components based on independent binomial trials for each year, whose mean is estimated from a single data point per year, have perfect fit. Finally, population estimates are in line with population counts, so that at least a systematic lack of fit appears unlikely. Formal parameter redundancy methods for IPMs have only been developed recently (Cole and McCrea 2016). Model selection was also conducted locally for model components when needed (e.g. MRR).

Independence between census and demographic datasets is a key assumption for forming the IPM joint likelihood by multiplying different likelihood components (Besbeas et al. 2009). Our datasets do not strictly meet this assumption, as some are obtained from the same colony areas and therefore include information from different life history aspects of the same individuals, also counted in the census. In practice, population counts can be considered independent because the island-wide counts are much larger than the sample of monitored birds. The impact of lack of independence in IPMs has yet to be thoroughly studied but it is likely to depend strongly on the nature of the dependency and degree of overlap in the number of shared individuals. The mixed results reported in the literature (c.f. simulation results of Besbeas et al. 2009; Abadi et al. 2010) may be caused by one of the datasets contributing most of the information for the parameter under study. Extending the framework to encompass several species may create new forms of dataset dependence across species.

Multi-species IPMs could also be extended to incorporate spatial aspects. For example, our multi-species synchrony IPM could be expanded to include other breeding colonies in the Northeast Atlantic with the same alcid community (or more generally, several populations of a set of species). Such a *multi-species multi-population IPM* would combine our idea with that of multi-population IPMs (Cave et al. 2010) and could allow the estimation of multi-population synchrony (Schaub et al. 2015).

Integrated population modelling makes explicit the relationship between changes in demographic rates and their impact on population fluctuations, and may bring insights into drastic population changes. Our extension of the IPM concept to encompass sympatric populations of several species allows at the same time the estimation of multi-species synchrony in a robust framework, and opens the door to further methodological developments.

## Electronic supplementary material

Below is the link to the electronic supplementary material.

Supplementary material 1 (pdf 160 KB)
