# Supplementary material for: Bringing It All Together: Multi-species Integrated Population Modelling of a Breeding Community
Source: J Agric Biol Environ Stat. 2017 Apr 18;22(2):140–60. doi: 10.1007/s13253-017-0279-4 (PMC7010376; doi:10.1007/s13253-017-0279-4)
Supplement: Supplementary file 1 — Supplementary material 1 (pdf 160 KB) [file 13253_2017_279_MOESM1_ESM.pdf]

Lahoz-Monfort, JJ, Harris, MP, Wanless, S, Freeman, SN, Morgan, BJT **Bringing it all together: multi-species Integrated Population Modelling of a breeding community**. *Journal of Agricultural, Biological and Environmental Statistics*.

APPENDIX: JAGS code to fit the multi-species synchrony IPM

species 1 = puffin; species 2 = murre; species 3 = razorbill

```
model {

#----------------------------------------------------------------------
#   PRODUCTIVITY
#----------------------------------------------------------------------

# priors ----------------------------------
for (i in 1:(T-1)){
    logit(rho1[i]) <- b0.rho1 + d.rho[i] + e1.rho[i]
    logit(rho2[i]) <- b0.rho2 + d.rho[i] + e2.rho[i]
    logit(rho3[i]) <- b0.rho3 + d.rho[i] + e3.rho[i]
      e1.rho[i] ~ dnorm (0,tau.e1.rho)
      e2.rho[i] ~ dnorm (0,tau.e2.rho)
      e3.rho[i] ~ dnorm (0,tau.e3.rho)
      d.rho[i] ~ dnorm (0,tau.d.rho)
}

b0.rho1 ~ dunif(-5,5)
b0.rho2 ~ dunif(-5,5)
b0.rho3 ~ dunif(-5,5)

se.d.rho ~ dunif(0,3)
var.d.rho <- se.d.rho*se.d.rho
tau.d.rho <-1/var.d.rho

se.e1.rho ~ dunif(0,3)
se.e2.rho ~ dunif(0,3)
se.e3.rho ~ dunif(0,3)
var.e1.rho <-se.e1.rho*se.e1.rho
var.e2.rho <-se.e2.rho*se.e2.rho
var.e3.rho <-se.e3.rho*se.e3.rho
tau.e1.rho <-1/var.e1.rho
tau.e2.rho <-1/var.e2.rho
tau.e3.rho <-1/var.e3.rho

# likelihood ------------------------------
for (i in 1:(T-1)){
      F1[i] ~ dbin(rho1[i],E1[i])
      F2[i] ~ dbin(rho2[i],E2[i])
      F3[i] ~ dbin(rho3[i],E3[i])
}

#----------------------------------------------------------------------
#   NON-BREEDING (GUILLEMOT ONLY)
#   B2[t] = probability of NOT skipping breeding
#----------------------------------------------------------------------

# priors ----------------------------------
for (i in 1:(T-1)){
    B2[i] ~ dunif(0,1)   #[uninformative]
}
# likelihood ------------------------------
for (i in 1:(T-1)){
      breed[i] ~ dbin(B2[i],monit[i])
}

#----------------------------------------------------------------------
#   BREEDING ADULTS MARK-RESIGHT DATA (ADULT SURVIVAL)
#----------------------------------------------------------------------

# Priors  ----------------------------------
for (i in 1:(T-2)){
      logit(sa1[i])<- b0.sa1 + d.sa[i] + e1.sa[i]
      logit(sa2[i])<- b0.sa2 + d.sa[i] + e2.sa[i]
      logit(sa3[i])<- b0.sa3 + d.sa[i] + e3.sa[i]
      e1.sa[i] ~ dnorm (0,tau.e1.sa)
```

```
        e2.sa[i] ~ dnorm (0,tau.e2.sa)
        e3.sa[i] ~ dnorm (0,tau.e3.sa)
        d.sa[i] ~ dnorm (0,tau.d.sa)
        # Year-dependent component of p
        pt1[i] ~ dnorm(0,1.0E-4)
        pt2[i] ~ dnorm(0,1.0E-4)
        pt3[i] ~ dnorm(0,1.0E-4)
        # p when not captured the previous occasion
        logit(p1[i]) <- pt1[i]
        logit(p2[i]) <- pt2[i]
        logit(p3[i]) <- pt3[i]
        # p when captured the previous occasion
        logit(pdep1[i]) <- pt1[i]+a1
        logit(pdep2[i]) <- pt2[i]+a2
        logit(pdep3[i]) <- pt3[i]+a3
}
a1 ~ dunif(-5,5)  # Prior for trap-dependence in p
a2 ~ dunif(-5,5)
a3 ~ dunif(-5,5)

b0.sa1 ~ dunif(-5,5)
b0.sa2 ~ dunif(-5,5)
b0.sa3 ~ dunif(-5,5)

se.d.sa ~ dunif(0,3)
var.d.sa <-se.d.sa*se.d.sa
tau.d.sa <-1/var.d.sa

se.e1.sa ~ dunif(0,3)
se.e2.sa ~ dunif(0,3)
se.e3.sa ~ dunif(0,3)
var.e1.sa <-se.e1.sa*se.e1.sa
var.e2.sa <-se.e2.sa*se.e2.sa
var.e3.sa <-se.e3.sa*se.e3.sa
tau.e1.sa <-1/var.e1.sa
tau.e2.sa <-1/var.e2.sa
tau.e3.sa <-1/var.e3.sa

# Multinomial likelihood --------------------
for (i in 1:(T-2)){
        m1[i,1:(T-1)] ~ dmulti(q1[i,],rel1[i])
        m2[i,1:(T-1)] ~ dmulti(q2[i,],rel2[i])
        m3[i,1:(T-1)] ~ dmulti(q3[i,],rel3[i])
}
# m-array cell probabilities ----------------
chi1[T-1]<-1    # chi (recursion for never seen)
chi2[T-1]<-1
chi3[T-1]<-1
for (j in 1:(T-2)){
        chi1[T-1-j]<- 1 - sa1[T-1-j]*(1-(1-p1[T-1-j])*chi1[T-1-j+1])
        chi2[T-1-j]<- 1 - sa2[T-1-j]*(1-(1-p2[T-1-j])*chi2[T-1-j+1])
        chi3[T-1-j]<- 1 - sa3[T-1-j]*(1-(1-p3[T-1-j])*chi3[T-1-j+1])
}
#  Cell probabilities
for (i in 1:(T-2)){
        # Cells in diagonal
        q1[i,i]<-pdep1[i]*sa1[i]
        q2[i,i]<-pdep2[i]*sa2[i]
        q3[i,i]<-pdep3[i]*sa3[i]
        # Cells above diagonal
        for (j in (i+1):(T-2)){
                logprod1[i,j,i]<-log(sa1[i]*(1-pdep1[i]))
                logprod2[i,j,i]<-log(sa2[i]*(1-pdep2[i]))
                logprod3[i,j,i]<-log(sa3[i]*(1-pdep3[i]))
                for (k in (i+1):(j-1)){
                        logprod1[i,j,k]<-log(sa1[k]*(1-p1[k]))
                        logprod2[i,j,k]<-log(sa2[k]*(1-p2[k]))
                        logprod3[i,j,k]<-log(sa3[k]*(1-p3[k]))
                }
                q1[i,j]<-p1[j]*sa1[j]*exp(sum(logprod1[i,j,i:(j-1)]))
                q2[i,j]<-p2[j]*sa2[j]*exp(sum(logprod2[i,j,i:(j-1)]))
                q3[i,j]<-p3[j]*sa3[j]*exp(sum(logprod3[i,j,i:(j-1)]))
        }
```

```
       # Empty cells below diagonal
       for (j in 1:(i-1)){
              q1[i,j]<-0
              q2[i,j]<-0
              q3[i,j]<-0}
       # Probability of an animal never seen
       q1[i,T-1] <- 1-sa1[i]*(1-(1-pdep1[i])*chi1[i+1])
       q2[i,T-1] <- 1-sa2[i]*(1-(1-pdep2[i])*chi2[i+1])
       q3[i,T-1] <- 1-sa3[i]*(1-(1-pdep3[i])*chi3[i+1])
}

#-----------------------------------------------------------------------------
#   GUILLEMOT CHICKS MARK-RESIGHT-RECOVERY (SURVIVAL)
#-----------------------------------------------------------------------------

# Priors -----------------------------------------------------------
for (t in 1:(T-1)){
    s1[t] ~ dunif(0,1)
}
sa2[T-1] ~ dunif(0,1)  # the rest are defined in the adult MR model part

s2 ~ dunif(0,1)
s35 ~ dunif(0,1

# for chicks from AREA A -----
for (t in 3:T){
    pC3A[t] ~ dunif(0,1)
}
for (t in 4:T){
    pC4A[t] ~ dunif(0,1)
}
for (t in 5:T){
    pC56A[t] ~ dunif(0,1)
}
for (t in 7:T){
    pCaA[t] ~ dunif(0,1)
}

# for chicks from AREA B -----
for (t in 3:17){
    pC3B[t] ~ dunif(0,1)
}
for (t in 4:18){
    pC4B[t] ~ dunif(0,1)
}
for (t in 5:20){
    pC56B[t] ~ dunif(0,1)
}
for (t in 7:T){
    pCaB[t] ~ dunif(0,1)
}

bC0 ~ dunif(-5,5)
bC1 ~ dunif(-5,5)
F5 ~ dunif(0,1)
F6 ~ dunif(0,1)
Fa ~ dunif(0,1)

# Parameter definition as function of age a and time t -----------
# true survival s[a,t] ---------------------
for (t in 1:(T-1)){
    s[1,t] <- s1[t]
}
# these can now be defined for all years, as info of sa2[ ] from MR dataset
for (t in 2:(T-1)){
    s[2,t] <- s2
}
for (t in 3:(T-1)){
    s[3,t] <- s35
}
for (t in 4:(T-1)){
    s[4,t] <- s35
}
for (t in 5:(T-1)){
    s[5,t] <- s35
}
for (a in 6:(T-1)){
```

```
    for (t in a:(T-1)){
        s[a,t] <- sa2[t]
    }
}
# resight probability pC_A[a,t] - chicks AREA A ---------------
for (t in 2:T) {
      pC_A[2,t] <- 0
}
for (t in 3:T) {
      pC_A[3,t] <- pC3A[t]
}
for (t in 4:T) {
      pC_A[4,t] <- pC4A[t]
}

for (a in 5:6) {
    for (t in a:T) {
        pC_A[a,t] <- pC56A[t]
    }
}
for (a in 7:A) {
    for (t in a:T) {
        pC_A[a,t] <- pCaA[t]
    }
}

# resight probability pC_B[a,t] - chicks AREA B ---------------
for (t in 2:T) {
      pC_B[2,t] <- 0
}
for (t in 3:17) {
      pC_B[3,t] <- pC3B[t]
}
for (t in 18:T) {
      pC_B[3,t] <- 0
}
for (t in 4:18) {
      pC_B[4,t] <- pC4B[t]
}
for (t in 19:T) {
      pC_B[4,t] <- 0
}
#----------
for (t in 5:19) {
      pC_B[5,t] <- pC56B[t]
}
for (t in 20:T) {
    pC_B[5,t] <- 0
}
for (t in 6:20) {
      pC_B[6,t] <- pC56B[t]
}
for (t in 21:T) {
    pC_B[6,t] <- 0
}
#----------
for (a in 7:A) {
    for (t in a:T) {
        pC_B[a,t] <- pCaB[t]
    }
}

# ring-recovery probability r(t) --------
for (t in 1:(T-1)){
    logit(r[t])<- bC0 + bC1*yr[t]
}
# fidelity F(t) -------------------------
for (a in 1:4){
    F[a] <- 1
}
F[5] <- F5
F[6] <- F6
for (a in 7:(A-1)){
    F[a] <- Fa
}
```

```
# Defining the probabilities - CHICKS AREA A ---------------------------

# chi[a,t]: probability animal seen for last time at age a at time t
for (a in 1:A){
    chiC0_A[a,T] <- 1
}
for (a in 1:A){
    for (t in a:(T-1)){
        chiC0_A[a,t] <- (1-r[t])*(1-s[a,t])+s[a,t]*chiC0_A[a+1,t+1]
    }
}
for (a in 1:A){
    chiC1_A[a,T] <- 1
}
for (a in 1:A){
    for (t in a:(T-1)){
        chiC1_A[a,t]<- (1-r[t])*(1-s[a,t])+s[a,t]*((1-F[a])*chiC0_A[a+1,t+1]+F[a]*(1-
pC_A[a+1,t+1])*chiC1_A[a+1,t+1])
    }
}

# Q[a,b,t]: probability animal of age a at time t is unobserved until age b+1
for (a in 1:(A-1)){
    for (t in a:(T-1)){
        Q00_A[a,a,t] <- s[a,t]
    }
}
for (a in 1:(A-2)){
    for (b in (a+1):(A-1)){
        for (t in a:(T-1+a-b)){
            Q00_A[a,b,t] <- s[a,t]*Q00_A[a+1,b,t+1]
        }
    }
}
for (a in 1:(A-1)){
    for (t in a:(T-1)){
        Q10_A[a,a,t] <- s[a,t]*(1-F[a])
    }
}
for (a in 1:(A-2)){
    for (b in (a+1):(A-1)){
        for (t in a:(T-1+a-b)){
            Q10_A[a,b,t] <- s[a,t]*((1-F[a])*Q00_A[a+1,b,t+1]+F[a]*(1-
pC_A[a+1,t+1])*Q10_A[a+1,b,t+1])
        }
    }
}
for (a in 1:(A-1)){
    for (t in a:(T-1)){
        Q11_A[a,a,t] <- s[a,t]*F[a]
    }
}
for (a in 1:(A-2)){
    for (b in (a+1):(A-1)){
        for (t in a:(T-1+a-b)){
            Q11_A[a,b,t] <- s[a,t]*F[a]*(1-pC_A[a+1,t+1])*Q11_A[a+1,b,t+1]
        }
    }
}

# O[a,b,t]: probability an animal observed at age a at time t remains
# unobserved until it is recaptured at age b+1
for (a in 1:(A-1)){
    for (b in a:(A-1)){
        for (t in a:(T-1+a-b)){
            O11_A[a,b,t] <- Q11_A[a,b,t]*pC_A[b+1,t+b-a+1]
        }
    }
}

# D[a,b,t]: probability an animal is recovered dead between ages b and b+1
# given it was last observed at age a at time t
for (a in 1:(A-1)){
    for (t in a:(T-1)){
        D1_A[a,a,t] <- (1-s[a,t])*r[t]
    }
}
```

```
for (a in 1:(A-1)){
    for (b in (a+1):(A-1)){
        for (t in a:(T-1+a-b)){
            D1_A[a,b,t] <- (1-s[b,t+b-a])*r[t+b-a]*(Q10_A[a,b-1,t]+Q11_A[a,b-1,t]*(1-pC_A[b,t+b-
a]))
        }
    }
}

# Defining the probabilities - CHICKS AREA B ---------------------------

# chi[a,t]: probability animal seen for last time at age a at time t
for (a in 1:A){
    chiC0_B[a,T] <- 1
}
for (a in 1:(T-1-14)){
    for (t in a:(a+14)){
        chiC0_B[a,t] <- (1-r[t])*(1-s[a,t])+s[a,t]*chiC0_B[a+1,t+1]
    }
}
for (a in (T-1-13):A){
    for (t in a:(T-1)){
        chiC0_B[a,t] <- (1-r[t])*(1-s[a,t])+s[a,t]*chiC0_B[a+1,t+1]
    }
}
for (a in 1:A){
    chiC1_B[a,T] <- 1
}
for (a in 1:(T-1-14)){
    for (t in a:(a+14)){
        chiC1_B[a,t]<- (1-r[t])*(1-s[a,t])+s[a,t]*((1-F[a])*chiC0_B[a+1,t+1]+F[a]*(1-
pC_B[a+1,t+1])*chiC1_B[a+1,t+1])
    }
}
for (a in (T-1-13):A){
    for (t in a:(T-1)){
        chiC1_B[a,t]<- (1-r[t])*(1-s[a,t])+s[a,t]*((1-F[a])*chiC0_B[a+1,t+1]+F[a]*(1-
pC_B[a+1,t+1])*chiC1_B[a+1,t+1])
    }
}

# Q[a,b,t]: probability animal of age a at time t is unobserved until age b+1
for (a in 1:(T-1-14)){
    for (t in a:(a+14)){
        Q00_B[a,a,t] <- s[a,t]
    }
}
for (a in (T-1-13):(A-1)){
    for (t in a:(T-1)){
        Q00_B[a,a,t] <- s[a,t]
    }
}
#---
for (a in 1:11){
    for (b in (a+1):12){
        for (t in a:(a+14)){
            Q00_B[a,b,t] <- s[a,t]*Q00_B[a+1,b,t+1]
        }
    }
    for (b in 13:(A-1)){
        for (t in a:(T-1+a-b)){
            Q00_B[a,b,t] <- s[a,t]*Q00_B[a+1,b,t+1]
        }
    }
}
for (a in 12:(A-2)){
    for (b in (a+1):(A-1)){
        for (t in a:(T-1+a-b)){
            Q00_B[a,b,t] <- s[a,t]*Q00_B[a+1,b,t+1]
        }
    }
}
#---
for (a in 1:(T-1-14)){
    for (t in a:(a+14)){
        Q10_B[a,a,t] <- s[a,t]*(1-F[a])
    }
```

```
}
for (a in (T-1-13):(A-1)){
    for (t in a:(T-1)){
        Q10_B[a,a,t] <- s[a,t]*(1-F[a])
    }
}
#---
for (a in 1:11){
    for (b in (a+1):12){
        for (t in a:(a+14)){
            Q10_B[a,b,t] <- s[a,t]*((1-F[a])*Q00_B[a+1,b,t+1]+F[a]*(1-
pC_B[a+1,t+1])*Q10_B[a+1,b,t+1])
        }
    }
    for (b in 13:(A-1)){
        for (t in a:(T-1+a-b)){
            Q10_B[a,b,t] <- s[a,t]*((1-F[a])*Q00_B[a+1,b,t+1]+F[a]*(1-
pC_B[a+1,t+1])*Q10_B[a+1,b,t+1])
        }
    }
}
for (a in 12:(A-2)){
    for (b in (a+1):(A-1)){
        for (t in a:(T-1+a-b)){
            Q10_B[a,b,t] <- s[a,t]*((1-F[a])*Q00_B[a+1,b,t+1]+F[a]*(1-
pC_B[a+1,t+1])*Q10_B[a+1,b,t+1])
        }
    }
}
#---
for (a in 1:(T-1-14)){
    for (t in a:(a+14)){
        Q11_B[a,a,t] <- s[a,t]*F[a]
    }
}
for (a in (T-1-13):(A-1)){
    for (t in a:(T-1)){
        Q11_B[a,a,t] <- s[a,t]*F[a]
    }
}
#---
for (a in 1:11){
    for (b in (a+1):12){
        for (t in a:(a+14)){
            Q11_B[a,b,t] <- s[a,t]*F[a]*(1-pC_B[a+1,t+1])*Q11_B[a+1,b,t+1]
        }
    }
    for (b in 13:(A-1)){
        for (t in a:(T-1+a-b)){
            Q11_B[a,b,t] <- s[a,t]*F[a]*(1-pC_B[a+1,t+1])*Q11_B[a+1,b,t+1]
        }
    }
}
for (a in 12:(A-2)){
    for (b in (a+1):(A-1)){
        for (t in a:(T-1+a-b)){
            Q11_B[a,b,t] <- s[a,t]*F[a]*(1-pC_B[a+1,t+1])*Q11_B[a+1,b,t+1]
        }
    }
}
#---

# O[a,b,t]: probability an animal observed at age a at time t remains
# unobserved until it is recaptured at age b+1
for (a in 1:12){
    for (b in a:12){
        for (t in a:(a+14)){
            O11_B[a,b,t] <- Q11_B[a,b,t]*pC_B[b+1,t+b-a+1]
        }
    }
    for (b in 13:(A-1)){
        for (t in a:(T-1+a-b)){
            O11_B[a,b,t] <- Q11_B[a,b,t]*pC_B[b+1,t+b-a+1]
        }
    }
}
```

```
for (a in 13:(A-1)){
    for (b in a:(A-1)){
        for (t in a:(T-1+a-b)){
            O11_B[a,b,t] <- Q11_B[a,b,t]*pC_B[b+1,t+b-a+1]
        }
    }
}
#---

# D[a,b,t]: probability an animal is recovered dead between ages b and b+1
# given it was last observed at age a at time t
for (a in 1:(T-1-14)){
    for (t in a:(a+14)){
        D1_B[a,a,t] <- (1-s[a,t])*r[t]
    }
}
for (a in (T-1-13):(A-1)){
    for (t in a:(T-1)){
        D1_B[a,a,t] <- (1-s[a,t])*r[t]
    }
}
#---
for (a in 1:11){
    for (b in (a+1):12){
        for (t in a:(a+14)){
            D1_B[a,b,t] <- (1-s[b,t+b-a])*r[t+b-a]*(Q10_B[a,b-1,t]+Q11_B[a,b-1,t]*(1-pC_B[b,t+b-
a]))
        }
    }
    for (b in 13:(A-1)){
        for (t in a:(T-1+a-b)){
            D1_B[a,b,t] <- (1-s[b,t+b-a])*r[t+b-a]*(Q10_B[a,b-1,t]+Q11_B[a,b-1,t]*(1-pC_B[b,t+b-
a]))
        }
    }
}
for (a in 12:(A-1)){
    for (b in (a+1):(A-1)){
        for (t in a:(T-1+a-b)){
            D1_B[a,b,t] <- (1-s[b,t+b-a])*r[t+b-a]*(Q10_B[a,b-1,t]+Q11_B[a,b-1,t]*(1-pC_B[b,t+b-
a]))
        }
    }
}
#---

# Multinomial likelihoods (chicks areas A & B) --------------------------

# for chicks AREA A
for (g in 1:sizeWA) {
     # single index, where a<-W[1,g] and t<-W[2,g]
     # Cell probabilities
     for (ii in 1:(T-WA[2,g])) {
        q2A[WA[3,g]-1+ii] <- O11_A[WA[1,g],ii-1+WA[1,g],WA[2,g]]
     }
     for (ii in (T-WA[2,g]+1):(2*(T-WA[2,g]))) {
        q2A[WA[3,g]-1+ii]<- D1_A[WA[1,g],ii-T+WA[2,g]-1+WA[1,g],WA[2,g]]
     }
     q2A[WA[4,g]] <- chiC1_A[WA[1,g],WA[2,g]]
     cellnums4A[WA[3,g]:WA[4,g]] ~ dmulti(q2A[WA[3,g]:WA[4,g]],relChicksA[WA[1,g],WA[2,g]])
}

# for chicks AREA B
for (g in 1:sizeWB) {
     # single index, where a<-W[1,g] and t<-W[2,g]
     # Cell probabilities
     for (ii in 1:(T-WB[2,g])) {
        q2B[WB[3,g]-1+ii] <- O11_B[WB[1,g],ii-1+WB[1,g],WB[2,g]]
     }
     for (ii in (T-WB[2,g]+1):(2*(T-WB[2,g]))) {
        q2B[WB[3,g]-1+ii]<- D1_B[WB[1,g],ii-T+WB[2,g]-1+WB[1,g],WB[2,g]]
     }
     q2B[WB[4,g]] <- chiC1_B[WB[1,g],WB[2,g]]
     cellnums4B[WB[3,g]:WB[4,g]] ~ dmulti(q2B[WB[3,g]:WB[4,g]],relChicksB[WB[1,g],WB[2,g]])
}
```

```
#-------------------------------------------------------------------------
#   POPULATION MODELS
#-------------------------------------------------------------------------
# Note fidelity and combined juvenile survival are confounded for P&R
# --> these are combined in a single variable "phicomb"
#-------------------------------------------------------------------------
# age of first breeding: d (=delay of d years to breed)
#-------------------------------------------------------------------------

# SPECIES1: PUFFINS ---------------------------------------------------

# Priors ---------------------------------
phicomb1 ~ dunif(0,1)
sd.N1 ~ dunif(0,15000)
var.N1 <-sd.N1*sd.N1
tau.N1 <-1/var.N1

# Initialisation of population model ----------
Ntmp1[1]~dnorm(12211,tau.N1)T(0,)
Ntmp1[2]~dnorm(13494,tau.N1)T(0,)
Ntmp1[3]~dnorm(14778,tau.N1)T(0,)
Ntmp1[4]~dnorm(16061,tau.N1)T(0,)
Ntmp1[5]~dnorm(17345,tau.N1)T(0,)
Ntmp1[6]~dnorm(18628,tau.N1)T(0,)
Ntmp1[7]~dnorm(19911,tau.N1)
N1[1]<-round(Ntmp1[1])
N1[2]<-round(Ntmp1[2])
N1[3]<-round(Ntmp1[3])
N1[4]<-round(Ntmp1[4])
N1[5]<-round(Ntmp1[5])
N1[6]<-round(Ntmp1[6])
N1[7]<-round(Ntmp1[7])

# Likelihood ---------------------------------
for (i in (d1+1):(T-1)){
    S1[i] ~ dbin(sa1[i-1],N1[i-1])
    combinedPr1[i] <- rho1[i-d1]*0.5*phicomb1*sa1[i-1]
    R1[i] ~ dbin(combinedPr1[i],N1[i-d1])
    N1[i] <- S1[i]+R1[i]
}

# Only 5 years of census (out of the initial period where can't estimate yet)
n1[1] ~ dnorm(N1[9],tau.N1)
n1[2] ~ dnorm(N1[15],tau.N1)
n1[3] ~ dnorm(N1[20],tau.N1)
n1[4] ~ dnorm(N1[25],tau.N1)
n1[5] ~ dnorm(N1[26],tau.N1)

# SPECIES2: GUILLEMOTS  ---------------------------------------------------

# Priors ---------------------------------
sd.N2 ~ dunif(0,5000)
var.N2 <-sd.N2*sd.N2
tau.N2 <-1/var.N2

# Initialisation of population model ----------
for (i in 1:d2) {
    Ntmp2[i]~dnorm(n2[i],tau.N2)
    N2[i]<-round(Ntmp2[i])
}

# Likelihood ---------------------------------
for (i in (d2+1):(T-1)){
    S2[i] ~ dbin(sa2[i-1],N2[i-1])
    combinedPr2[i-d2] <- B2[i-d2]*rho2[i-d2]*0.5*s1[i-d2]*s[2,i-5]*s[3,i-4]*s[4,i-3]*s[5,i-
2]*F5*s[6,i-1]*F6
    R2[i] ~ dbin(combinedPr2[i-d2],N2[i-d2])
    N2[i] <- S2[i]+R2[i]
    n2[i] ~ dnorm(N2[i],tau.N2)
}

# SPECIES3: RAZORBILLS ---------------------------------------------------

# Priors ---------------------------------
phicomb3 ~ dunif(0,1)
sd.N3 ~ dunif(0,5000)
var.N3 <-sd.N3*sd.N3
```

```
tau.N3 <-1/var.N3

# Initialisation of population model ----------
for (i in 1:d3) {
    Ntmp3[i]~dnorm(n3[i],tau.N3)
    N3[i]<-round(Ntmp3[i])
}

# Likelihood ----------------------------------
for (i in (d3+1):(T-1)){
    S3[i] ~ dbin(sa3[i-1],N3[i-1])
    combinedPr3[i] <- rho3[i-d3]*0.5*phicomb3*sa3[i-1]
    R3[i] ~ dbin(combinedPr3[i],N3[i-d3])
    N3[i] <- S3[i]+R3[i]
    n3[i] ~ dnorm(N3[i],tau.N3)
}

## Adult population growth rates --------------------------------
## [can be calculated here but done offline to save memory of the resulting file]
#for (i in 1:(T-2)){
#    lambda1[i]<-N1[i+1]/N1[i]
#    lambda2[i]<-N2[i+1]/N2[i]
#    lambda3[i]<-N3[i+1]/N3[i]
#}

#---------------------------------------------------------------
# ICCs: synchrony parameters
# [can be calculated here but done offline to save memory of the resulting file]
#---------------------------------------------------------------
#ICC.rho.1 <- var.d.rho/(var.e1.rho+var.d.rho)
#ICC.rho.2 <- var.d.rho/(var.e2.rho+var.d.rho)
#ICC.rho.3 <- var.d.rho/(var.e3.rho+var.d.rho)

#ICC.sa.1 <- var.d.sa/(var.e1.sa+var.d.sa)
#ICC.sa.2 <- var.d.sa/(var.e2.sa+var.d.sa)
#ICC.sa.3 <- var.d.sa/(var.e3.sa+var.d.sa)

}
```
